# Supplementary material for: Impact of cervical screening by human papillomavirus genotype: Population-based estimations
Source: PLoS Med. 2023 Oct 27;20(10):e1004304. doi: 10.1371/journal.pmed.1004304 (PMC10637721; doi:10.1371/journal.pmed.1004304)
Supplement: S1 Appendix — (DOCX) [file pmed.1004304.s002.docx]

S1 Appendix: Organized cervical screening program and registry in Sweden

Since the 1970s, population-based cervical screening with cytology has been recommended and implemented every three years for women at the age of 23-49 years and every five years for women at the age of 50-60 years in Sweden (some regions implemented up to age of 64 years). Human papillomavirus (HPV) test was introduced as a screening tool in 2015 [1], with updated recommendation that women at the age of 23-29 years receive primarily cytology whereas women at the age of 30-64 years receive primarily HPV test with cytology as triage. Screening interval remains 3-year for the ages of 23-49 years and 5-year for the ages of 50-64 years. The implementation started from January 2017 in region of Stockholm, and gradually rolled out to other regions in the following years. The recently updated guideline in 2022 recommends HPV-based screening for all ages, with 5-year interval for ages of 23-49 years and 7-year interval for ages of 50-70 years [2]. Women with a positive screening result are subsequently referred to colposcopy and biopsy for histopathological diagnosis of cervical cancer or precancerous lesion. All data concerning cervical screening, precancerous lesion, and cervical cancer are included in the Swedish National Cervical Screening Registry (NKCx in Swedish acronym), with nationwide coverage since 1993-1995 [3]. As reported by NKCx, screening coverage reaches at around 80% in the past 10 years [4].

References:

1. National Board of Health and Welfare (Socialstyrelsen). Screening for cervical cancer. [Accessed 15 Sept 2023]. Available: https://www.socialstyrelsen.se/globalassets/sharepoint-dokument/artikelkatalog/nationella-screeningprogram/2022-2-7758-bilaga1.pdf

2. Livmoderhalscancer – screening med HPV-test. In: Socialstyrelsen [Internet]. 7 Jun 2021 [Accessed 29 Jun 2023]. Available: https://www.socialstyrelsen.se/kunskapsstod-och-regler/regler-och-riktlinjer/nationella-screeningprogram/slutliga-rekommendationer/livmoderhalscancer/

3. Elfström KM, Sparén P, Olausson P, Almstedt P, Strander B, Dillner J. Registry-based assessment of the status of cervical screening in Sweden. J Med Screen. 2016;23: 217–226. doi:10.1177/0969141316632023

4. The Swedish National Cervical Screening Registry. Swedish National Cervial Screening Registry: Annual Report 2021. [Accessed 28 Feb 2022]. Available: https://nkcx.se/templates/_rsrapport_2021.pdf
