# Supplementary material for: Impact of cervical screening by human papillomavirus genotype: Population-based estimations
Source: PLoS Med. 2023 Oct 27;20(10):e1004304. doi: 10.1371/journal.pmed.1004304 (PMC10637721; doi:10.1371/journal.pmed.1004304)
Supplement: S1 Table — (DOCX) [file pmed.1004304.s003.docx]

S1A Table: Impact numbers and bootstrap confidence intervals (CI) for detecting and preventing one cervical cancer case by human papillomavirus (HPV) type, ages 23-60 years

|  | Numbers needed to screen | | | | Numbers needing follow-up | | | |
| --- | --- | --- | --- | --- | --- | --- | --- | --- |
|  | To **detect** one case | | To **prevent** one case | | To **detect** one case | | To **prevent** one case | |
|  | Point Estimate | 95%CI | Point Estimate | 95%CI | Point Estimate | 95%CI | Point Estimate | 95%CI |
| HPV 16 | 13,885 | 13,055-14,711 | 5,527 | 5,076-6,054 | 371 | 348-393 | 147 | 135-161 |
| HPV 18 | 37,280 | 33,208-42,531 | 32,125 | 22,857-51,703 | 356 | 317-406 | 307 | 218-494 |
| HPV 31 | 202,547 | 146,665-303,641 | 120,247 | 68,863-308,693 | 3,208 | 2,323-4,810 | 1,905 | 1,091-4,890 |
| HPV 33 | 245,200 | 179,845-360,818 | 83,884 | 58,160-142,879 | 1,421 | 1,042-2,092 | 486 | 337-828 |
| HPV 35 | 5,100,308 | 2,179,269-∞* | 267,045 | 172,517-548,947 | 17,120 | 7,315-∞ | 896 | 579-1,842 |
| HPV 39 | 1,008,712 | 661,911-1,839,113 | 220,334 | 122,873-666,337 | 4,314 | 2,831-7,866 | 942 | 525-2,850 |
| HPV 45 | 116,091 | 96,915-141,237 | 43,655 | 31,616-71,019 | 1,760 | 1,469-2,141 | 662 | 479-1,076 |
| HPV 51 | 5,239,990 | 2,308,080-∞ | ∞ | ∞ | 59,133 | 26,046-∞ | ∞ | ∞ |
| HPV 52 | 445,906 | 271,521-938,375 | 380,757 | 191,942-6,585,713 | 6,386 | 3,888-13,439 | 5,453 | 2,749-94,323 |
| HPV 56 | 871,466 | 497,358-2,164,234 | 479,556 | 259,624-3,043,325 | 7,112 | 4,059-17,662 | 3,913 | 2,118-24,837 |
| HPV 58 | 2,533,751 | 1,241,130-10,888,885 | 230,175 | 144,792-458,546 | 14,692 | 7,196-63,140 | 1,334 | 839-2,658 |
| HPV 59 | 1,252,334 | 782,321-2,749,462 | 1,339,680 | 404,361-∞ | 4,103 | 2,563-9,008 | 4,389 | 1,324-∞ |
| HPV 66 | 7,065,580 | 2,833,427-∞ | 852,629 | 390,679-5,011,452 | 35,589 | 14,272-∞ | 4,294 | 1,967-25,242 |
| HPV 68 | 18,162,183 | 5,773,130-∞ | 783,010 | 349,387-7,980,928 | 31,878 | 10,133-∞ | 1,374 | 613-14,008 |

*Infinity

S1B Table: Impact numbers and bootstrap confidence intervals (CI) for detecting and preventing one cervical cancer case, by human papillomavirus (HPV) type and age group

|  | Numbers needed to screen | | | | Numbers needing follow-up | | | |
| --- | --- | --- | --- | --- | --- | --- | --- | --- |
|  | To **detect** one case | | To **prevent** one case | | To **detect** one case | | To **prevent** one case | |
|  | Point Estimate | 95%CI | Point Estimate | 95%CI | Point Estimate | 95%CI | Point Estimate | 95%CI |
| Age 23-30 years | | | | | | | | |
| HPV16 | 24,518 | 21,594-28,004 | 4,747 | 3,938-5,967 | 1,491 | 1,313-1,703 | 289 | 239-363 |
| HPV18 | 49,036 | 39,801-63,603 | 50,908 | 19,931-∞ | 1,160 | 941-1,504 | 1,204 | 471-∞ |
| HPV45 | 261,524 | 148,879-620,331 | 62,546 | 24,843-∞ | 8,393 | 4,778-19,909 | 2,007 | 797-∞ |
| Medium* | 261,524 | 148,407-634,510 | 52,212 | 23,846-∞ | 22,972 | 13,036-55,734 | 4,586 | 2,095-∞ |
| Lower** | 1,176,857 | 465,071-∞*** | 221,345 | 55,665-∞ | 89,457 | 35,352-∞ | 16,825 | 4,231-∞ |
| Age 31-40 years | | | | | | | | |
| HPV16 | 11,409 | 10,649-12,311 | 4,808 | 3,995-5,953 | 336 | 314-363 | 142 | 118-176 |
| HPV18 | 29,589 | 25,532-35,019 | 14,811 | 10,174-26,154 | 274 | 237-325 | 137 | 94-243 |
| HPV45 | 95,775 | 72,789-134,040 | 36,764 | 20,663-146,401 | 1,486 | 1,129-2,079 | 570 | 321-2,271 |
| Medium* | 125,498 | 93,095-191,629 | 53,493 | 25,675-866,866 | 5,388 | 3,997-8,228 | 2,297 | 1,102-37,220 |
| Lower** | 330,859 | 203,323-748,260 | 64,862 | 31,990-859,134 | 12,920 | 7,940-29,220 | 2,533 | 1,249-33,550 |
| Age 41-50 years | | | | | | | | |
| HPV16 | 20,674 | 18,705-22,886 | 4,959 | 4,254-5,983 | 285 | 258-316 | 68 | 59-83 |
| HPV18 | 45,342 | 38,071-55,833 | 23,522 | 14,899-47,345 | 237 | 199-291 | 123 | 78-247 |
| HPV45 | 107,841 | 80,469-154,232 | 25,137 | 15,802-47,587 | 1,011 | 755-1,447 | 236 | 148-446 |
| Medium* | 128,714 | 96,265-190,954 | 30,049 | 19,054-66,135 | 3,343 | 2,500-4,960 | 780 | 495-1,718 |
| Lower** | 221,674 | 148,151-391,686 | 52,585 | 28,535-196,848 | 5,237 | 3,500-9,253 | 1,242 | 674-4,650 |
| Age 51-60 years | | | | | | | | |
| HPV16 | 25,527 | 22,658-28,613 | 5,114 | 4,700-5,540 | 292 | 259-327 | 58 | 54-63 |
| HPV18 | 71,477 | 57,740-96,061 | 22,367 | 18,540-28,673 | 278 | 225-374 | 87 | 72-112 |
| HPV45 | 127,637 | 93,962-194,494 | 43,635 | 33,114-63,046 | 1,054 | 776-1,605 | 360 | 273-520 |
| Medium* | 170,182 | 120,256-263,689 | 15,460 | 13,191-18,828 | 3,887 | 2,747-6,023 | 353 | 301-430 |
| Lower** | 297,819 | 186,817-597,664 | 47,338 | 35,029-68,422 | 6,178 | 3,875-12,397 | 982 | 727-1,419 |

*Medium oncogenic types include HPV 31, 33, 52, 58 (etiological fraction>2% according to IARC’s data [4])

**Lower oncogenic types include HPV 35, 39, 51, 56, 59, 66, 68 (etiological fraction<2% according to IARC’s data [4])

***Infinity
