# Supplementary material for: Impact of cervical screening by human papillomavirus genotype: Population-based estimations
Source: PLoS Med. 2023 Oct 27;20(10):e1004304. doi: 10.1371/journal.pmed.1004304 (PMC10637721; doi:10.1371/journal.pmed.1004304)
Supplement: S1 Protocol — (PDF) [file pmed.1004304.s004.pdf]

REGIONALA ETIKPRÖVNINGSNÄMNDEN • STOCKHOLM • EPN

PROTOKOLL 2011/4:7
2011-08-24

Sammanträde i Stockholm

Avdelning 4

Ordförande
Annika Marcus

Ledamöter med vetenskaplig kompetens
Sigurd Vitols, vetenskaplig sekreterare *(klinisk farmakologi)*
Guro Gafvelin *(molekylär immunologi)*
Tommy Linné *(barnmedicin), deltog inte i ärende 2011/1203*
Erik Sundström *(geriatrik)*
Britt Gustafsson *(barnmedicin), deltog inte i ärende 2011/91*
Erling Löfsjögård Nilsson *(klinisk fysiologi)*
Anna-Berit Ransjö Arvidson *(vårdvetenskap, internationell hälsa)*
Erik Näslund *(kirurgi)*

Ledamöter som företräder allmänna intressen
Rut Andersson
Roland Håkansson
Kjell Haglund
Åke Reisnert
Gunnar Skoglund

Administrativ sekreterare
Lena Creutzer Waldersten

§ 1 Ordföranden förklarar sammanträdet öppnat.

§ 2 Den administrativa sekreteraren anmäler att den vetenskaplige sekreteraren sedan föregående möte den 8 juni 2011 fattat beslut i 49 ärenden som avser ändring av godkännande.

§ 3 Ansökningar om etisk granskning av forskningsprojekt, se **Bilaga**.

§ 4 Ordföranden förklarar sammanträdet avslutat och meddelar att nästa sammanträde i avdelning 4 äger rum den 21 september 2011.

Sigurd Vitols, protokollförare
Vetenskaplig sekreterare

Annika Marcus
Ordförande

------------ Utdrag ur protokoll från sammanträde 24 augusti 2011 i avdelning 4 ----------

Dnr 2011/1026-31/4
Föredragande:
Anna-Berit Ransjö Arvidsson

Sökande: Karolinska Institutet
Behörig företrädare: Kamila Czene
Projekt: Orsaker till cervixcancer i Sverige trots organiserad gynekologisk cellprovtagning. Fortsättningsstudie.
Forskare som genomför projektet: Pär Sparén

BESLUT

Nämnden godkänner forskningen.

Beslut expedierat till behörig företrädare, forskare och i förekommande fall även sponsor

Att utdraget överensstämmer med originalet intygas:

Lena Creutzer Waldersten, administrativ sekreterare

expedierat 2011-09-01

ANSÖKAN OM ETIKPRÖVNING

se anvisning sid. 16

Uppgifter som fylls i av den regionala etikprövningsnämnden

Ankomstdatum:

Dnr:

Avgift inbetald datum:

Begäran om komplettering av ansökan:

Ansökan komplett:

Begäran om ytterligare information:

Begärd information inkommen:

Expeditionsdatum:

Beslutsdatum:

Uppgifter som fylls i av sökanden

Till Regionala etikprövningsnämnden i: Stockholm
(Den regionala etikprövningsnämnd till vars upptagningsområde forskningshuvudmannen hör, se www.forskningsetikprovning.se)

Projekt

Ange en beskrivande titel på svenska för lekmän, utan sekretesskyddad information.<br>Ange också i förekommande fall projektets identitet, projektets/forskningsplanens (protokollets eller prövningsplanens) nummer, version, datum osv.

Orsaker till cervixcancer i Sverige trots organiserad gynekologisk cellprovtagning. Fortsättningsstudie.

Projektnummer/identitet: Version nummer:

Ansökan avser (gäller även vid begäran om rådgivande yttrande):

forskning där endast en forskningshuvudman deltar (5000 kr) ☒

forskning där fler än en huvudman deltar (16000 kr) ☐

forskning där mer än en forskningshuvudman deltar, men där samtliga forskningspersoner eller forskningsobjekt enligt 4 § lagen (2003:460) om etikprövning av forskning som avser människor, har ett omedelbart samband endast med en av forskningshuvudmännen (5000 kr) ☐

endast behandling av personuppgifter (5000 kr) ☐

forskning som gäller klinisk läkemedelsprövning (16000 kr) ☐

ändring av tidigare godkänd ansökan (enligt 4 §) (2000 kr) ☐

Om nämnden finner att studien/forskningsprojektet inte faller inom lagens för etikprövning tillämpningsområde önskas ett rådgivande yttrande

Ja: ☒ Nej: ☐

1. Information om forskningshuvudman m.m.

1:1 Sökande forskningshuvudman

Ansökan om etikprövning av forskning skall göras av forskningshuvudmannen. *Med forskningshuvudman avses en statlig myndighet eller en fysisk eller juridisk person i vars verksamhet forskningen utförs.* Inom staten utförs forskning främst vid lärosätena, men även vid vissa andra myndigheter, som t.ex. Brottsförebyggande rådet och Socialstyrelsen. Kommuner och landsting kan vara forskningshuvudmän, liksom privaträttsliga juridiska personer.

Namn: Institutionen för medicinsk epidemiologi och biostatistik
Karolinska Institutet

Adress: Box 281
171 77 Stockholm

1:2 Behörig företrädare

Behörig företrädare för forskningshuvudmannen (t.ex. prefekt, enhetschef, verksamhetschef). Forskningshuvudmännen bestämmer själva, genom interna arbets- och delegationsordningar eller genom fullmakt, vem som är behörig att företräda forskningshuvudmannen. Bifoga kopia av sådan handling.

Namn: Kamila Czene

Tjänstetitel: Professor, t.f. prefekt

Adress: Institutionen för medicinsk epidemiologi och biostatistik , Karolinska institutet

1:3 Forskare som är huvudansvarig för genomförandet av projektet (kontaktperson)

Namn: Pär Sparén

Adress: Institutionen för medicinsk epidemiologi och biostatistik, Karolinska Institutet

E-postadress: Par.Sparen@ki.se

Telefon: 08-524 861 02

Mobiltelefon: 070-794 46 74

1:4 Plats

Plats/er där projektet skall genomföras (ange inrättning/ar, institution/er, klinik/er etc.).

Inst f Medicinsk Epidemiologi och Biostatistik Karolinska Institutet

1:5 Andra medverkande

Övriga deltagande forskningshuvudmän samt forskare ansvariga att lokalt genomföra projektet (kontaktpersoner) skall anges i bilaga (namn, adresser).

Bengt Andrae, Center for Research and Development, Uppsala universitet/landstinget Gävleborg, Joakim Dillner, MEB/KI, Lena Silfverdal KK UNN, Björn Strander OC Göteborg, Walter Ryd Patologen SU Sahlgrenska, Sven Törnberg OC Stockholm

1:6 Vid läkemedelsprövning

Ansökan om tillstånd har insänts till Läkemedelsverket.

Ansökan inlämnad (datum):

Tillstånd erhållits ☐

1:7 Vid viss genetisk forskning

Anmälan till *Datainspektionen* om förhandskontroll av behandling av personuppgifter om genetiska anlag som framkommit efter genetisk undersökning (10 § första stycket 2 personuppgiftsförordningen (1998:1191).

Inlämnad (datum): Kommer att inlämnas efter godkänd etikprövning ☐

2. Uppgifter om projektet

2:1 Sammanfattande beskrivning av forskningsprojektet (programmet)

Beskrivningen skall kunna förstås av nämndens lekmän. Undvik därför terminologi som kräver specialkunskaper. Ange bakgrund och syfte för studien samt den (de) vetenskapliga frågeställning (ar) som man söker svar på. Ange de viktigaste undersökningsvariablerna. Ange vilka kunskapsvinster projektet kan förväntas ge och betydelsen av dessa. Ange om det är en registerstudie, uppdragsforskning etc. För fackmän avsedd detaljerad information i protokoll eller forskningsplan _skall_ bifogas som bilaga. En utförligare beskrivning över genomförandet *avsedd för lekmän* kan vid behov bifogas den för fackmän avsedda obligatoriska forskningsplanen.

Livmoderhalscancer är en förebyggbar sjukdom där man genom att spåra och avlägsna förstadier i form av cellförändringar kan hindra insjuknande i cancer. De svenska cellprovtagningsprogrammen är uppbyggda landstingsvis och sinsemellan ganska olika vad gäller organisation, åldersgrupper som kallas m.m. Fastän man under en fyrtioårig verksamhet reducerat sjukligheten i l betydligt, så avlider fortfarande lika många kvinnor årligen i denna sjukdom i landet som i trafikolyckor.
För att kunna prioritera arbetet med att förbättra programmen krävs en bättre kunskap om vilka brister i programmen som gör att kvinnor fortfarande får livmoderhalscancer och dör i sjukdomen. Detta är huvudsyftet med föreliggande forskningsprogram, som avses bli den vetenskapliga grunden för förbättrad prevention av livmoderhalscancer. Studien genomförs som en nationell, befolkningsbaserad fall—kontrollstudie. Alla nya invasiva livmoderhalscancerfall i Sverige från 1999 och framåt, (cirka 450 fall per år), identifieras och karakteriseras samt jämförs med kvinnor i samma ålder, som ej fått livmoderhalscancer, avseende hur programmen fungerat, hur och när cellprov tagits samt hur och när behandling av cellförändringar skett. Resultaten av detta projekt

kommer att direkt kunna peka på förebyggbara brister i det svenska cellprovtagningsprogrammen och deras relativa betydelse. Eftersom projektet genomförs i nära samarbete med dem som ansvarar för screeningprogrammen och arbetar med uppföljning och behandling finns direkta kanaler ut i verksamheten, Vetenskaplig analys av bristernas betydelse i förening med utvärdering av resultaten av insatserna är de enda sätten att säkerställa att optimal effekt fås ut av de resurser som investerats i denna omfattande, och nationella, cancerförebyggande verksamhet.

2:2 Vilken primär vetenskaplig frågeställning ligger till grund för projektets utformning

Om projektet kan karakteriseras som en hypotesprövning, ange den primära och eventuellt sekundära hypotesen. Hänvisning till mer detaljerad information för fackmän kan ske till bifogat protokoll eller forskningsplan enligt 2:1

Att vetenskapligt undersöka varför incidensen av livmoderhalscancer inte har sjunkit mer efter 40 år av landsomfattande cytologisk screening. Att med utgångspunkt i vetenskapligt definierade orsaker till varför livmoderhalscancer kan uppstå, trots screeningprogram, identifiera strategier för ett förbättrat screeningprogram mot livrnoderhalscancer.

Att undersöka prognos efter diagnos av invasiv livmoderhalscancer för kvinnor som har deltagit respektive inte deltagit i screening.

Att undersöka hur tidig diagnos av invasiv livmoderhalscancer påverkar möjligheten att föda fullgångna barn.

Att undersöka hur dysplasibehandling påverkar risken för prematuritet (för tidigt födda barn).

2:3 Redogör för resultat från relevanta djurförsök

För viss, främst medicinsk, forskning ange skälen till att djurförsök inte utförts.

Ej relevant

2:4 Redogör översiktligt för undersökningsprocedur, datainsamling och datas karaktär

Av beskrivningen skall framgå hur studien planeras genomföras. Beskriv insamlade datas karaktär. Hur säkerställs datas tillförlitlighet (t.ex. kvalitetskontroll/monitorering)? - Vid enkäter och intervjuer skall beskrivas tillvägagångssätt och t.ex. frågors innehåll och hur slutsatser dras. Enkäter och skattningsskalor skall bifogas. - För medicinsk forskning skall anges t.ex. typer av ingrepp, mätmetoder, antal besök, tidsåtgång vid varje försök, doser och administrationssätt för eventuella läkemedel och/eller isotoper, blodprovsmängd (även ackumulerad mängd vid multipla försök). Ange även om och på vilket sätt undersökningsprocedur m.m. skiljer sig från klinisk rutin. Ange proceduren för att ge den eventuella behandling efter studiens slut, som kan erfordras. Ange procedur för insamling av biologiskt material. Redogör för datakällor och procedurer vid behandling av personuppgifter. För mer detaljerad information kan hänvisning ske till bilagt protokoll eller forskningsplan enligt 2:1.

Studien avses att genomföras som en nationell, populationsbaserad fall — kontrollstudie för att följa förändringar över tid. Alla primära invasiva fall av cervixcancer i Sverige under åren 1999 och framåt (totalt cirka 450 fall per år) identifieras det nationella cancerregistret. Härifrån inhämtas också information om diagnosdatum, tumörtyp, diagnosgrund, kliniskt stadium (där denna uppgift finns), dödsfall och dödsorsak, samt uppgift om tumören var ett obduktionsfynd. Alla fall av cancer uteri UNS identifieras likaså från det nationella cancerregistret. Diagnostiska histopatologiska preparat från fall av cervixcancer och cancer uteri UNS efterfrågas för eftergranskning. Vid eftergranskningen avgörs om diagnosen är invasiv cervixcancer eller ej och vilken dess histopatologiska tumörtyp är. För fall där kliniskt stadium vid cervixcancerdiagnos ej registrerats kommer uppgiften att efterfrågas från journalhandlingar.
Från det svenska befolkningsregistret identifieras kontroller för varje cancerfall, födda samma år, i livet och utan cervixcancer eller cancer uteri UNS vid tidpunkten för fallets diagnos. För den information som hämtas ur cytologiregister avses alla levande kvinnor, som ej har fått cervixcancer och födda samma år som fallet, utgöra kontroller. För information som eftersöks manuellt identifieras fem kontroller för varje fall enligt ovan. För fall och kontroller inhämtas information om screeninghistoria avseende alla registrerade cellprov och HPV-tester, all cervixpatologi, information om behandlingshistorik från kvinnokliniker och gyn-onkologiska kliniker, samt tidpunkt för konisation. Information om dödsdatum och dödsorsak från dödsorsaksregistret, utvandring från befolkningsregistret, samt barnafödande och graviditetsufall från det medicinska födelseregistret inhämtas och för fall och kontroller.

Analys av data sker sedan i två steg och resultaten presenteras som statistiska sammanställningar där inga enskilda individer kan identifieras:
1. Deskriptiv statistik: åldersspecifik incidens i cervixcancer och dess fördelning på kliniskt stadium vid diagnos, histologisk tumörtyp och andel intervallcancer. Denna statistik presenteras för hela landet, samt för varje landsting separat.
2. Relativa och absoluta risker att insjukna i cervixcancer beroende av screening— och behandlingshistoria, samt andelen cervixcancer som har orsakats av brister inom screeningprogrammet.

2:5 Redogör för om insamlat biologiskt material kommer att förvaras i en biobank

Med biobank avses biologiskt material från en eller flera människor som samlas och bevaras tills vidare eller för en bestämd tid och vars ursprung kan härledas till den eller de människor från vilka materialet härrör.

Redogör för var och hur prover som skall sparas förvaras, kodningsprocedurer och villkor för utlämnande av prover. Observera att i förekommande fall skall anmälan av biobank ske till Socialstyrelsen enligt lagen (2002:297) om biobanker i hälso- och sjukvården m.m.

Diagnostiska preparat kommer att insamlas från patologiska laboratorier för omgranskning. Dessa återlämnas efter genomförd omgranskning.

2:6 Redovisa tillgång till nödvändiga resurser under hela projektets genomförande

Ange vilka som har ansvaret (prefekt, verksamhetschef eller motsvarande) för forskningspersonernas säkerhet vid alla enheter/kliniker där patienter ingår samt att erforderliga ekonomiska och personella resurser finns tillgängliga. Intyg från dessa skall bifogas.

2:7 Journalföring, registrering och hantering av data

Redogör för hur undersökningsprocedurer och eventuella ingrepp journalförs. Ange hur registrering och behandling av resultaten skall gå till. Om materialet skall kodas, ange proceduren, vem som förvarar kodlistor och vem eller vilka som har tillgång till dem, var de förvaras, hur länge samt om materialet kommer att anonymiseras eller förstöras. Används band- och videoinspelningar? Vilken tillgänglighet har datamaterialet? Hur förvaras det? Hur erhålls erforderligt sekretesskydd?

Alla resultat redovisas som statistiska sammanställningar där enskilda individer ej kan identifieras. Personnummer används endast för att kunna länka rätt information till rätt individ och tas bort innan analys av data. Alla analyser sker vid inst. för medicinsk epidemiologi och biostatistik, KI. Institutionen har ett skalskydd och alla datorer är säkrade innanför en s.k. brandvägg, vilken förhindrar åtkomst utifrån. Varje dator har en personlig användarkod och ett lösenord som byts var 5:e vecka. För alla som arbetar i forskargruppen och vid institutionen gäller sekretesslagen. Efter analyser kommer ingen information att kunna spåras till enskilda individer.

2:8 Redogör för tidigare erfarenheter (egna och/eller andras) av den använda proceduren, tekniken eller behandlingen

Särskilt angeläget är att redovisning av risker för komplikationer görs tydliga och i förekommande fall med angivande av relevanta publikationer. Om ansökan avser fortsättning eller uppföljning av tidigare projekt, ange diarienummer samt datum för beslut av tidigare godkänd ansökan. Vid nya läkemedelsbehandlingar av patienter bör anges hur många patienter (med aktuell eller annan åkomma) som tidigare erhållit föreslagen eller högre dosering samt hur långa behandlingsperioder som studerats.

Forskargruppen har långvarig erfarenhet av registerbaserade epidemiologiska studier och befolkningsbaserade fall-kontrollstudier för att underöka orsaker till livmoderhalscancer, prevention av sjukdomen, samt prognos efter diagnos. En första audit baserad på alla fall av livmoderhalscancer i Sverige 1999-2001 publicerades nyligen.

3. Uppgifter om forskningspersoner

3:1 Hur görs urvalet av forskningspersoner

Med forskningsperson avses en levande människa som forskningen avser.

Ange urvalskriterier (inklusion och exklusion). På vilket sätt kommer forskaren i kontakt med/får kännedom om lämpliga forskningspersoner? Ange om rekrytering sker från (egna, andras) tidigare eller pågående studier. Om annonsering sker, skall annonsmaterialet insändas som bilaga. Om t.ex. barn, eller personer som tillfälligt eller permanent inte är kapabla att ge ett eget informerat samtycke skall tillfrågas om deltagande i projektet, skall detta särskilt motiveras. Om vissa grupper (t.ex. kvinnor, barn eller äldre) utesluts från deltagande i projektet skall detta särskilt motiveras.

Alla fall av livmoderhalscancer i Sverige från 1999 och framåt (ungefär 450 fall per år) , samt Sveriges kvinnliga befolkning i motsvarande åldrar som kontroller, vilka hämtas från det svenska befolkningsregistret.

3:2 Ange relationen mellan forskare/försöksledare och forskningspersonerna

☐ Behandlare (t.ex. läkare, psykolog, sjukgymnast) - forskningsperson (t.ex. patient, klient)

☐ Kursgivare (lärare) - student

☐ Arbetsgivare - anställd

☐ Annan relation. Beskriv:

3:3 Redogör för det statistiska underlaget för studiepopulationens (ernas)/ undersökningsmaterialets(-ens) storlek

Redovisa en statistisk styrka, så kallad "power"-beräkning eller motsvarande överväganden för tydliggörande av studiens möjligheter att besvara frågeställningarna.

För det enkla fallet att alla riskfaktorer för cervixcancer mäts och analyseras individuellt har lägsta detekterbara relativa risk för cervixcancer beräknats vid $\alpha = 0.05$ och 80% power. Dessa beräkningar har gjorts för olika värden av riskfaktorprevalens, samt för olika antal fall och kontroller för att ge en uppfattning om effekten av stratifierade analyser. För hela materialet kan vi detektera relativa risker ner till 1,2 vid optimal exponering för riskfaktorer bland kontrollerna (50%), men även vid väsentligt lägre riskfaktorexponering (10% - 25%) är lägsta detekterbara relativa risk fortfarande mycket låg (1,2 – 1,3). Vid stratifierade analyser på små grupper om 150 fall blir lägsta detekterbara relativa risk väsentligt högre (1,8 – 2,2), medan den för 300 – 500 fall håller sig väl under 2,0. Sammanfattningsvis kan sägas att våra möjligheter att detektera även små skillnader i relativ risk mellan fall och kontroller är mycket goda och att vi också kommer att ha god möjlighet att stratifiera analyserna för att t.ex. studera olika tumörtyper eller kliniska stadier separat.

3:4 Kan forskningspersonerna komma att inkluderas i flera studier samtidigt eller i nära anslutning till denna studie

Ange om forskningspersonerna kan inkluderas samtidigt i flera studier eller i nära anslutning till denna studie. Ange i så fall projekttitel, forskningshuvudman, forskare som genomför studien (kontaktperson) samt diarienummer (om känt) för de övriga studierna. När avslutades ett eventuellt tidigare deltagande?

Nej

3:5 Vilket försäkringsskydd finns för de forskningspersoner som deltar i projektet

Det åligger forskningshuvudmannen att kontrollera att befintliga försäkringar täcker eventuella skador som kan

uppkomma.

Ej relevant

3:6 Vilken ekonomisk ersättning eller andra förmåner utgår till de forskningspersoner som deltar i projektet och när betalas ersättningen ut (Utförligare beskrivning kan lämnas i bilaga)

Ersättning för obehag och besvär. Belopp (före skatt):

Ersättning för förlorad arbetsinkomst ☐ Ja ☐ Nej

Reseersättning ☐ Ja ☐ Nej

Befrielse från kostnader för läkemedel ☐ Ja ☐ Nej

Befrielse från andra kostnader. Vilka?

Andra förmåner. Vilka?

När betalas ersättningen ut?

Ingen ersättning betalas ut ☒

4. Information och samtycke

4:1 Proceduren för och innehållet i den *information* som lämnas då forskningspersoner tillfrågas om deltagande

Beskriv hur och när information ges och vad den innehåller. Vem informerar? Normalt skall en kortfattad och lättförståelig skriftlig information ges. Denna skriftliga information skall bifogas ansökan. Om ingen eller ofullständig information ges, måste skälen för detta noggrant anges.<br><br>***Forskningspersonen skall informeras om***<br>- den övergripande planen för forskningen<br>- syftet med forskningen<br>- de metoder som kommer att användas<br>- de följder och risker som forskningen kan medföra<br>- vem som är forskningshuvudman och kontaktperson<br>- att deltagandet i forskningen är frivilligt och<br>- forskningspersonens rätt att när som helst avbryta sin medverkan.

De kvinnor som deltar i cytologisk screening är medvetna om att detta är en cancerförebyggande verksamhet och förutsätter i allmänhet att deras prover ordnas i dataregister liksom att uppgifterna används för att dra konsekvenser av utfallet.

4:2 Hur och från vem inhämtas *samtycke*

Beskriv proceduren; vem som frågar, när detta sker och hur samtycket dokumenteras. Utförlig redovisning är särskilt viktig då barn eller personer med nedsatt beslutskompetens ingår i studien, likaså vid studier av en grupp (grupper), t.ex. föreningar, organisationer, företag, kyrkosamfund och församlingar eller arbetet i en skolklass.

Det är inte möjligt att inhämta samtycke om deltagande för alla kvinnor i studien, eftersom de är flera miljoner till antalet. Den information som inhämtas i denna studie finns redan sammanställd regionalt. Identifikation med personnummer behövs endast för att länka rätt information till rätt individ och kommer att tas bort så snart all information är

inhämtad. Avsikten med studien är att göra kvantitativa analyser av orsaker till att livmoderhalscancer uppkommer trots förekomst av screeningprogram. Så snart den relevanta informationen är inhämtad behöver vi inte veta vilka kvinnorna är. Vi kommer ej att kontakta kvinnorna i någon del av detta projekt och deras uppföljning/behandling kommer ej att påverkas av studien. Det är också angeläget att samtyckesordningen blir densamma som vid tidigare audit, eftersom data annars inte kan jämföras och ett viktigt syfte med studien (uppmäta eventuella kvalitetsförändringar över tid) därmed inte kan uppfyllas

5. Forskningsetiska överväganden

5:1 Redogör för risker som deltagandet kan medföra samt möjliga komplikationer

Detta kan vara t.ex. smärta, obehag eller integritetsintrång som projektet innebär eller kan innebära.
Har åtgärder vidtagits för att förebygga de risker som sägs ovan? Vilken beredskap finns att hantera dessa komplikationer? Ange metoder som kommer att användas för att efterforska, registrera och rapportera oönskade händelser.

Sammanställning av cytologiska screeningdata satt i relation till cervixcancersjuklighetet (histologi, tumörstadium och behandlingsdata) skulle kunna tolkas som ett integritetsintrång. Dessa uppgifter finns emellertid i de flesta fall redan samlade inom samma sjukvårdsdistrikt, ofta vid samma laboratorium.
Uppföljningen av screeningprogramrnet inklusive uppföljning av inträffade cancerfall finns föreslaget bl.a. i SoS—rapport 1998:15 och Socialstyrelsens föreskrifter. Liknande procedurer förutsättes i andra EU-länder.
De kvinnor som deltar i cytologisk screening är medvetna om att detta är en cancerförebyggande verksamhet och förutsätter i allmänhet att deras prover ordnas i dataregister liksom att uppgifterna används för att dra konsekvenser av utfallet. Kvinnorna förväntar sig också att datasystemen bevakar när det är dags att ta nästa prov.

Personidentifierade data är en förutsättning för att kunna följa upp enskilda cancerfall liksom för att kunna agera vid avvikande provsvar, eller för att kalla individer och grupper som ej detagit inom önskat provtagnings intervall.
Sammanfattningsvis måste risken för integritetsintrång betraktas som mycket ringa i förhållande till den nytta som detta forskningsprogram kommer att medföra för alla kvinnor i landet som är sexuellt aktiva och därmed löper risk att drabbas av livmoderhalscancer.Det är också angeläget att samtyckesordningen blir densamma som vid tidigare audit, eftersom data annars inte kan jämföras och ett viktigt syfte med studien (uppmäta eventuella kvalitetsförändringar över tid) därmed inte kan uppfyllas

5:2 Redogör för förutsebar nytta för de forskningspersoner som ingår i projektet

I framtiden kan alla sexuellt aktiva kvinnor dra nytta av förbättringar av screeningprogrammet, inklusive de som ingår i denna studie.

5:3 Gör en egen värdering av förhållandet risk – nytta för de forskningspersoner som deltar

Alla kvinnor som deltar i studien kan ha nytta av ett förbättrat screeningprogram, samt undvika överdiagnostik och överbehandling efter diagnos. Risken för ev. integritetsintrång måste betraktas som begränsad eftersom de aktuella uppgifterna redan finns tillgängliga regionalt. Sammantaget anses därför nyttan överstiga risken.

5:4 Identifiera och precisera vilka etiska problem t.ex. risk – nytta i ett vidare perspektiv som kan uppstå inom eller genom projektet

I ett vidare perspektiv måste nyttan för alla sexuellt aktiva kvinnor i landet att undvika livmoderhalscancer i framtiden, eller att överleva en diagnos av invasiv livmoderhalscancer, vägas mot den begränsade risken för integritetsintrång för de som ingår i studien. Bedömningen blir då att nyttan vida överstiger risken.

6. Redovisning av resultaten

6:1 Hur garanteras forskningshuvudmannen och medverkande forskare tillgång till data (anges vid t.ex. uppdragsforskning) och vem ansvarar för databearbetning och rapportskrivning

Vid uppdragsforskning anges hur forskningshuvudmannen och medverkande forskare garanteras tillgång till data och vem som ansvarar för databearbetning och rapportskrivning.

Databearbetning sker vid MEB, KI. Pär Sparén ansvarar tillsammans med biostatistiker vid samma institution för databearbetning. Bengt Andrae ansvarar tillsammans med Pär Sparén och Joakim Dillner för författande av vetenskapliga artiklar.

6:2 Hur kommer resultaten att göras offentligt tillgängliga Kommer studien att insändas för publicering i tidskrift eller publiceras på annat sätt

Ange i vilken form resultaten planeras offentliggöras samt tidsplan för detta.

Publicering av resultat i vetenskapliga artiklar.

6.3 På vilket sätt garanteras forskningspersonernas rätt till integritet när materialet offentliggörs/publiceras

Beskriv procedurer eller metod för avidentifiering/anonymisering. Redovisas endast resultat på statistisk gruppnivå?

Data avidentifieras efter länkning mellan registren och redovisas endast på gruppnivå.

7. Redovisning av ekonomiska förhållanden och beroendeförhållanden

7:1 Vid uppdragsforskning

Ange uppdragsgivaren t.ex. vid klinisk läkemedelsprövning.

Namn: Kontaktperson:

Adress: Telefon/mobiltelefon:

Ange uppdragsgivarens relation till forskningshuvudmannen/medverkande forskare, t.ex. anställningsförhållande.

7:2 Redovisa eventuella ekonomiska överenskommelser med uppdragsgivare eller andra finansiärer (namn, belopp)

Vid klinisk läkemedelsprövning kan hänvisning ske till ingånget avtal med sjukvårdshuvudmannen eller genom uppgift om föreslagen ersättning enligt överenskommelsen mellan Landstingsförbundet och LIF som bifogas. Separata överenskommelser med den/de som skall genomföra forskningen skall också redovisas. Om överenskommelserna inte är klara i sin helhet vid tidpunkten för ansökan skall belopp för studien/ersättning till kliniken/genomföraren och vad ersättningen skall täcka alternativt belopp per forskningsperson anges här. Vid studier där fler än en forskningshuvudman deltar skall principerna för och storleksordningen för ersättning för studien i sin helhet anges.

7:3 Redovisa forskningshuvudmannens och medverkande forskares intressen/tillgångar

Redovisa de som kan tänkas påverka tilltron till objektiviteten i genomförande och rapportering (t.ex. aktieinnehav eller konsultuppdrag i finansierande företag).

Ej aktuellt

8. Förteckning över bilagor

Dokument som, i tillämpliga fall, skall bifogas om inte motsvarande information finns i blanketten har markerats med x. Markera de bilagor som skickas in med denna ansökan.

Insänd med ansökan	Bil nr	Beskrivning	Klinisk läkemedels prövning	Annan forskning
☐	1<br>p 1:5	Deltagande forskningshuvudmän och medverkande forskare (kontaktpersoner) vid forskning där mer än en forskningshuvudman deltar	x	x
☐	2<br>p 2:1	För fackmän avsedd projekt/forskningsplan (protokoll), vid behov även för lekmän avsedd bilaga	x	x
☐	3<br>p 3:1	Annonsmaterial för rekrytering av forskningspersoner	x	x
☐	4<br>p 4:1	Skriftlig information till dem som tillfrågas	x	x
☐	5<br>p 2:4	Enkät, frågeformulär	x	x
☐	6	Gemensam EU-blankett (gäller fr.o.m. den 1 maj 2004), gäller även vid ändring	x	
☐	7	Sammanfattning av protokollet på svenska	x	
☐	8	Prövarhandbok alt. bipacksedel/produktresumé	x	
☒	9<br>p 2:6	Intyg från verksamhetschef/motsv. om resurser	x	x
☒	10	CV för forskare (samma som p 1:3) med huvudansvar för genomförande (redovisa forskarens kompetens)	x	x
☐	11<br>p 3:6	Beskrivning av ersättning till forskningspersoner	x	x
☐	12<br>p 7:1<br>p 7:2	Överenskommelser med uppdragsgivare/finansiär om t.ex. anställningsförhållanden, bidrag/ersättning till prövningsplats, sjukvårdshuvudman, forskningshuvudman eller forskare	x	x
☐	13	Tillstånd från strålskyddskommitté eller motsvarande	x	x

9. Undertecknande

Behörig företrädare för sökande forskningshuvudman enligt p 1:2.

Ort: Stockholm Datum: 20110622

Signatur Czene

Namnförtydligande: Kamila Czene

Undertecknad forskare som genomför projektet (kontaktperson) enligt p 1:3 intygar härmed att forskningen kommer att genomföras i enlighet med ansökan.

Ort: Stockholm Datum: 20110622

Signatur

Namnförtydligande: Pär Sparén

Anvisning för ansökan

Denna ansökningsblankett används vid ansökan om etikprövning enligt lagen (2003:460) om etikprövning av forskning som avser människor. Den är avsedd att användas för all

slags forskning där godkännande skall inhämtas från en etikprövningsnämnd. Blanketten skall användas även vid begäran om rådgivande yttrande enligt 2 § förordning (2003:616) med instruktion för regionala etikprövningsnämnder. Beroende på vilken forskning som ansökan gäller kommer de uppgifter som efterfrågas nedan att ha olika relevans. Därmed varierar också kravet på utförlighet i redovisningen av dessa. Markera på formuläret när uppgiften inte berör det aktuella projektet. Ansökan skall ifyllas så att den blir lättläst, dvs. den skall inte vara handskriven och inte skriven med liten stil och kort radavstånd. Blanketten skall skrivas på svenska.

Observera att en ansökan aldrig är komplett (och därmed kan behandlas) förrän blanketten är korrekt ifylld och avgiften är betald.
Ansökan med bilagor insändes i ett original och 16 kopior!

Om denna blankett
Allt som skall fyllas i är markerat grått. Övrig förklarande text är låst och får inte ändras. De grå fälten expanderar allteftersom du skriver, det finns alltså obegränsat utrymme för din text. Vid utskrift syns enbart texten du fyllt i och ej de grå fälten som syns på skärmen, det är därför viktigt att kontrollera att allt verkligen är ifyllt. När du är klar skriver du ut blanketten, kompletterar med erforderliga underskrifter samt bilagor och skickar till den regionala etikprövningsnämnd till vars upptagningsområde forskningshuvudmannen hör, se www.forskningsetikprovning.se.

Karolinska Institutet

Institutionen för medicinisk epidemiologi
och biostatistik
Pär Sparén
Par.Sparen@ki.se

2012-06-04

Regionala etikprövningsnämnden i Stockholm
2012 -06- 05
Dnr: 2012/1028/32
Handl:

Regionala Etikprövningsnämnden i Stockholm

Komplettering av etiskt tillstånd för projektet "Orsaker till cervixcancer i Sverige trots organiserad gynekologisk cellprovtagning. Fortsättningsstudie" (dnr 2011/1026-31/4).

I nämnda projekt kommer vi att samla in histologiska preparat för alla fall av cervixcancer i Sverige från 2002 och framåt för eftergranskning. Cervixcancer orsakas nästan alltid av HPV, men för att avgöra vilken typ av HPV-virus som cancern orsakats av behöver vi göra nya snitt i de befintliga tumörbitar som finns sparade på de olika patologilaboratorierna samt testa dessa för HPV. Denna information är viktig för att kunna avgöra effekten av HPV-vaccination och HPV-baserad screening i Sverige. All testning av material utföres under kod och information om HPV-typ kopplas inte till identifierad person. Information om HPV-typ i cervixtumörerna kommer heller inte att återföras till patienterna eller behandlande läkare eftersom patienterna redan är behandlade för sin sjukdom och informationen om HPV-typ saknar betydelse för den kliniska handläggningen.

Med vänliga hälsningar,

Pär Sparén
Professor

GODKÄNNS Dat. 120620

Sigurd Vitols
Vetenskaplig sekreterare
Regionala etikprövningsnämnden
i Stockholm

Karolinska Institutet

2012-06-04

Regionala etikprövningsnämnden i Stockholm

2012 -06- 05

Dnr: 2012/1028/32

Handl:

Institutionen för medicinisk epidemiologi
och biostatistik
Pär Sparén
Par.Sparen@ki.se

Regionala Etikprövningsnämnden i Stockholm

Komplettering av etiskt tillstånd för projektet "Orsaker till cervixcancer i Sverige trots organiserad gynekologisk cellprovtagning. Fortsättningsstudie" (dnr 2011/1026-31/4).

I nämnda projekt kommer vi att samla in histologiska preparat för alla fall av cervixcancer i Sverige från 2002 och framåt för eftergranskning. Cervixcancer orsakas nästan alltid av HPV, men för att avgöra vilken typ av HPV-virus som cancern orsakats av behöver vi göra nya snitt i de befintliga tumörbitar som finns sparade på de olika patologilaboratorierna samt testa dessa för HPV. Denna information är viktig för att kunna avgöra effekten av HPV-vaccination och HPV-baserad screening i Sverige. All testning av material utföres under kod och information om HPV-typ kopplas inte till identifierad person. Information om HPV-typ i cervixtumörerna kommer heller inte att återföras till patienterna eller behandlande läkare eftersom patienterna redan är behandlade för sin sjukdom och informationen om HPV-typ saknar betydelse för den kliniska handläggningen.

Med vänliga hälsningar,

Pär Sparén
Professor

GODKÄNNS Dat. 120620

Sigurd Vitols
Vetenskaplig sekreterare
Regionala etikprövningsnämnden
i Stockholm

Protocol 2011 4:7

Ethical Review Agency in Stockholm: Division 4

Chairperson

Annika Marcus

Board members with scientific competence

8 members listed

Board members representing the public

5 members listed

Administraative secretary

Lena Creutzer Waldensten

§1 Meeting opened

§2 Information that Changes in projects have been approved for 49 projects.

§3. Ethical vetting. Decisions in appendix

§4. Meeting closed. Next meeting on 21 September

Signed by scientific secretary Sigurd Vitols and Annika Marcus, Chairperson

Excerpt from Appendix regarding 2011-1026-31/4

Institute

Applicant

Project Title

DECISION

The Board approves the research

Google translate of Ethical Permission 2011-1036-31/4

APPLICATION FOR ETHICAL PERMISSION

Title; Causes of cervical cancer in Sweden despite organized cervical screening. A continuation study.

.

Applicant institute: Karolinska Institutet, Dept. of Medical Epidemiology & Biostatistics. Box 281, 171 77 Stockholm

Authorized signatory: Kamila Czene, Professor, Acting Head of Department

Karolinska Institutet, Dept. of Medical Epidemiology & Biostatistics.

Main Principal Investigator:

Pär Sparen, Karolinska Institutet, Dept. of Medical Epidemiology & Biostatistics. Par.sparen@ki.se

Place: Karolinska Institutet, Dept. of Medical Epidemiology & Biostatistics

Other Principal Investigators:

Bengt Andrae, Center for Research and Development, Uppsala university/landstinget Gävleborg, Joakim Dillner, MEB/KI, Lena Silfverdal KK UNN, Björn Strander OC Gothenburg, Walter Ryd Patologen SU Sahlgrenska, Sven Törnberg OC Stockholm

Introduction;

Cervical cancer is a preventable disease where, by tracking and removing precursors in the form of cell changes, cancer can be prevented. The Swedish cell sampling programs are structured by county council and between them quite differently in terms of organization, age groups called etc. Although morbidity has been significantly reduced in the course of a forty-year operation, as many women still die annually from this disease in the country as from traffic

accidents. In order to be able to prioritize the work to improve the programs, a better knowledge is required of which shortcomings in the programs mean that women still get cervical cancer and die from the disease. This is the main aim of the present research program, which is intended to be the scientific basis for improved prevention of cervical cancer. The study is conducted as a national, population-based case–control study. All new invasive cervical cancer cases in Sweden from 1999 onwards, (around 450 cases per year), are identified and characterized and compared with women of the same age, who have not had cervical cancer, regarding how the programs functioned, how and when cell samples were taken and how and when treatment of cell changes occurred. The results of this project will be able to directly point to preventable deficiencies in the Swedish cell sampling program and their relative importance. Since the project is carried out in close collaboration with those responsible for the screening programs and working with follow-up and treatment, there are direct channels out into the business. Scientific analysis of the significance of the shortcomings in combination with evaluation of the results of the efforts are the only ways to ensure that optimal effect is obtained from the resources invested in this extensive, and national, cancer prevention activity.

Major hypotheses under study:

To scientifically investigate why the incidence of cervical cancer has not decreased more after 40 years of nationwide cytological screening.

Based on scientifically defined reasons why cervical cancer can occur, despite screening programs, to identify strategies for an improved screening program against cervical cancer.

To examine prognosis after diagnosis of invasive cervical cancer for women who have and have not participated in screening.

To investigate how early diagnosis of invasive cervical cancer affects the possibility of giving birth to full-term children.

To investigate how dysplasia treatment affects the risk of prematurity (children born too early).

Google translate of Ethical Permission 2011-1036-31/4

Study plan:

The study is intended to be conducted as a national, population-based case–control study to monitor changes over time. All primary invasive cases of cervical cancer in Sweden during the years 1999 and onwards (a total of approximately 450 cases per year) are identified in the national cancer registry. From here, information is also collected on date of diagnosis, tumor type, basis for diagnosis, clinical stage (where this information is available), death and cause of death, as well as information on whether the tumor was an autopsy finding. All cases of cancer uteri UNS are also identified from the national cancer registry. Diagnostic histopathological preparations from cases of cervical cancer and uterine cancer UNS are requested for follow-up review. During the follow-up examination, it is determined whether the diagnosis is invasive cervical cancer or not and what its histopathological tumor type is. For cases where clinical stage at diagnosis of cervical cancer has not been registered, the information will be requested from medical records. From the Swedish population register, controls are identified for each cancer case, born in the same year, alive and without cervical cancer or uterine cancer UNS at the time of the case's diagnosis. For the information retrieved from cytology registers, all living women, who have not had cervical cancer and born in the same year as the case, are intended to constitute controls. For information searched manually, five controls are identified for each case as above. For cases and controls, information on screening history regarding all registered pap smears and HPV tests, all cervical pathology, information on treatment history from women's clinics and gynecological oncology clinics, and time of conization is obtained. Information on date of death and cause of death from the cause of death register, emigration from the population register, as well as childbirth and pregnancy loss from the medical birth register is obtained and for cases and controls. Analysis of the data then takes place in two stages and the results are presented as statistical summaries where no individual individuals can be identified: 1. Descriptive statistics: age-specific incidence in cervical cancer and its distribution by clinical stage at diagnosis, histological tumor type and proportion of interval cancer. These statistics are presented for the entire country, as well as for each county council separately. 2. Relative and absolute

risks of contracting cervical cancer depending on screening and treatment history, as well as the proportion of cervical cancer that has been caused by deficiencies within the screening program.

Will biological materials be biobanked?

Diagnostic specimens will be collected from pathology laboratories for review. These are returned after a re-examination has been completed.

Data management:

All results are reported as statistical summaries where individual individuals cannot be identified. Social security number is only used to be able to link the right information to the right individual and is removed before analysis of the data. All analyzes take place at inst. for medical epidemiology and biostatistics, Kl. The department has a shell protection and all computers are secured inside a so-called firewall, which prevents access from the outside. Each computer has a personal user code and a password that is changed every 5 weeks. The confidentiality act applies to everyone who works in the research group and at the department. After analyses, no information will be traceable to individuals.

Previous experiences of the approach:

The research group has long-term experience with registry-based epidemiological studies and population-based case-control studies to investigate causes of cervical cancer, prevention of the disease, and prognosis after diagnosis. A first audit based on all cases of cervical cancer in Sweden 1999-2001 was recently published.

Selection of subjects for study:

All cases of cervical cancer in Sweden from 1999 onwards (approximately 450 cases per year), as well as Sweden's female population of corresponding ages as controls, which are taken from the Swedish population register.

Statistical power:

For the simple case that all risk factors for cervical cancer are measured and analyzed individually, the lowest detectable relative risk for cervical cancer has been calculated at $\alpha = 0.05$ and 80% power. These calculations have been made for different values of risk factor prevalence, as well as for different numbers of cases and controls to give an idea of the effect of stratified analyses. For the entire material, we can detect relative risks down to 1.2 at optimal exposure to risk factors among controls (50%), but even at substantially lower risk factor exposure (10% - 25%) the lowest detectable relative risk is still very low (1.2 – 1,3). In stratified analyzes on small groups of 150 cases, the lowest detectable relative risk becomes substantially higher (1.8 – 2.2), while for 300 – 500 cases it remains well below 2.0. In summary, it can be said that our opportunities to detect even small differences in relative risk between cases and controls are very good and that we will also have a good opportunity to stratify the analyzes in order to e.g. study different tumor types or clinical stages separately.

Do you plan overlapping studies

No

Are the patients insured:

Not relevant

Are the patients paid for participation:

No

How are the patients informed:

The women who participate in cervical screening are aware that this is a cancer prevention activity and generally assume that their samples are arranged in data registers as well as that the data is used to draw consequences from the outcome.

Google translate of Ethical Permission 2011-1036-31/4

How do you obtain consent:

It is not possible to obtain consent for participation from all the women in the study, as they number several million. The information collected in this study is already compiled regionally. Identification with social security number is only needed to link the correct information to the correct individual and will be removed as soon as all information is collected. The purpose of the study is to make quantitative analyzes of reasons why cervical cancer occurs despite the existence of screening programs. Once the relevant information is obtained, we do not need to know who the women are. We will not contact the women in any part of this project and their follow-up/treatment will not be affected by the study. It is also important that the consent procedure be the same as in previous audits, because otherwise the data cannot be compared and an important purpose of the study (measuring possible quality changes over time) cannot therefore be fulfilled

What are the risks to the participants?

Compilation of cytological screening data set in relation to cervical cancer incidence (histology, tumor stage and treatment data) could be interpreted as a violation of privacy. However, in most cases this information is already collected within the same healthcare district, often at the same laboratory. The follow-up of the screening program, including the follow-up of cancer cases that have occurred, is proposed, among other things. in SoS report 1998:15 and the National Board of Health and Welfare's regulations. Similar procedures are assumed in other EU countries. The women who participate in cytological screening are aware that this is a cancer prevention activity and generally assume that their samples are arranged in data registers as well as that the data is used to draw consequences from the outcome. The women also expect the computer systems to monitor when it is time to take the next test. Personally identifiable data is a prerequisite for being able to follow up individual cancer cases as well as for being able to act in the event of abnormal test results, or to call individuals and groups that did not sample within the desired sampling interval. In summary, the risk of breach of privacy must be considered very small in relation to the benefit that this research program will bring to all

women in the country who are sexually active and thus at risk of contracting cervical cancer. It is also important that the consent procedure be the same as in previous audits , because otherwise the data cannot be compared and an important purpose of the study (measuring possible quality changes over time) thus cannot be fulfilled

Are there benefits to the participants?

In the future, all sexually active women may benefit from improvements in the screening program, including those included in this study.

What is your evaluation of the risk to benefit ratio?

All women who participate in the study can benefit from an improved screening program, as well as avoid overdiagnosis and overtreatment after diagnosis. The risk of possible privacy breach must be considered limited as the relevant data is already available regionally. Overall, the benefit is therefore considered to exceed the risk

Are there ethical risks in a broader perspective?

In a broader perspective, the benefit for all sexually active women in the country to avoid cervical cancer in the future, or to survive a diagnosis of invasive cervical cancer, must be weighed against the limited risk of invasion of privacy for those included in the study. The assessment then becomes that the benefit far exceeds the risk.In a wider perspective, the benefit for all sexually active women in the country to avoid cervical cancer in the future, or to survive a diagnosis of invasive cervical cancer, must be weighed against the limited risk of privacy infringement for those included in the study. The assessment then becomes that the benefit far exceeds the risk

Describe the data management. Who is responsible for writing the publications?

Data processing takes place at MEB, KI. Pär Sparén is responsible together with biostatisticians at the same department for data processing. Bengt Andrae is

responsible together with Pär Sparén and Joakim Dillner for writing scientific articles.

How will the results be made public?

Publication of results in scientific articles.

How is the integrity of individuals protected in the publications?

The data is de-identified after linking between the registers and is only reported at group level.

Do you have economic interests in the study?

No

Instructions regarding appendices.

Signed by Pär Sparen and Kamila Czene

General instructions on how to fillout the form

Department of Medical Epidemiology and biostatistics

Pär Sparén

Par.Sparen@ki.se

Regional Ethics Review Board in Stockholm

Amendment to ethical permission for the project "Causes of cervical cancer in Sweden despite organized gynecological pap smears. A continuation study" (dnr 2011/1026-31/4). In the mentioned project, we will collect histological specimens for all cases of cervical cancer in Sweden from 2002 onwards for follow-up examination. Cervical cancer is almost always caused by HPV, but in order to determine the type of HPV virus that caused the cancer, we need to make new incisions in the existing tumor pieces that are stored at the various pathology laboratories and test these for HPV. This information is important to be able to determine the effect of HPV vaccination and HPV-based screening in Sweden. All testing of material is carried out under code and information on HPV type is not linked to an identified person. Information about the HPV type in the cervical tumors will also not be returned to the patients or attending physicians because the patients have already been treated for their disease and the information about the HPV type has no significance for the clinical management.

Sincerely, Pär Sparén Professor

Stamp of the Regional Ethical Review Agency of Stockholm

APPROVED

Date 12-06-20

Signature of Sigurd Vitols

Scientific Secretary of the Regional Ethical Review Agency of Stockholm

Deviation from original protocol:

When estimating age-specific cervical cancer incidence by screening, the starting year changed from 1999 to 2004, which was due to that in this specific study the screening history was defined as 10 years prior to cancer diagnosis, and the screening registry NKCx did not reach nearly full coverage before 1993.
