# Supplementary material for: Impact of cervical screening by human papillomavirus genotype: Population-based estimations
Source: PLoS Med. 2023 Oct 27;20(10):e1004304. doi: 10.1371/journal.pmed.1004304 (PMC10637721; doi:10.1371/journal.pmed.1004304)
Supplement: Attachment — Submitted filename: Response_to_Editors_reviewers_20230630.docx [file pmed.1004304.s006.docx]

Responses to editors’ and reviews’ comments

ACADEMIC EDITOR COMMENTS

1. It's not that well-written in my opinion. In particular, I found it very hard to follow the methods - it is really hard to know when actual empirical data are being used and presented and when they are based on assumptions. I would like to see this much more clearly in the revised version. I've made some specific comments below.

*Reply: We have substantially revised the text to more clearly emphasize that we systematically use empirical data. Abstract, introduction, method section and Figure 1 are revised to more clearly detail the study population and the empirical data sources. The study has 3 assumptions which were previously discussed in the supplementary material. We now moved them the to the main text in the method section under each relevant paragraph: HPV type-specific prevalences in the population, calender priod stability, and cervical cancer risk between screened and unscreened population.*

2. Title: ‘impact numbers’ not meaningful – this term should be revised in the title and throughout the manuscript.
*Reply: As requested, we have changed the title to “Impact of different types of Human Papillomavirus in cervical screening: Population-based estimations”*

3. Abstract: will probably need to be rewritten, based on reviewer 4’s review – but as it stands, it’s hard to understand

*Reply: we have re-written the abstract.*

4. Materials and methods, p4: This section starts ‘This study was based on the entire population
of women live [sic] in Sweden from 1990s to 2019…’ But some data sources are for sub-populations and estimates are presumably extrapolated. Could the authors a) make it clearer from the beginning that data sources are not all for the whole population and b) say what the age range is that defines ‘all women’?

*Reply: We have re-written the study population description. The impact was estimated using i) the effect of screening in the population of women aged 25-80, resident in Sweden during 2004-2011 (N=3,568,938); ii) HPV type-specific prevalences among cervical cancers in Sweden in 2002-2011 (N=4,254); iii) HPV 16/18/Other prevalences in the population-based HPV screening program (N=656,607); and iv) exact HPV genotyping in a population-based cohort (n=12,527).*

*We now describe each one of the populations that provide the required input data clearly in both the abstract and in a re-drawn study flow chart (Figure 1) and revised methods section. All sources of data were individual level, identifiable data.*

5. Materials and methods, p5: ‘by screening history’ – this seems to be applying an assumption, rather than being based on women’s actual screening history. Is this correct? If so, can it be more explicit? On p4, it says that each woman’s screening history was retrieved – so how were these data used? It would actually be very helpful to have a table that lists the actual data sources and says how these data are then used.

*Reply: It is indeed the actual screening history of each woman. The Swedish National Cervical Screening Registry contains all individual-level screening records in the country and was linked on individual-level with the Total Population Registry and National Cancer Registry. We have elucidated it more clearly in the re-written abstract, the methods section and in the re-drawn study flow chart (figure 1).*

6. Tables, figures: Age groups should be reported consistently. E.g. Fig 1, first box, Swedish women aged 23-84; left had side box has 25-84; middle boxes – there are numbers stratified by different age groups – please explain in legend. Table 1 start at <30 – is this from age 23 or 25? And >=65, is this up to 84 or all over that age? Table 4, why are preventable cases 61-80 included in age group 51-60? If oldest age group is 84 elsewhere, why not here?
*Reply: The intention is to not have any age restrictions. However, screening starts at age 23 and there are very few observations before age 25. Similarly screening hardly have any effect above age 80, simply because screening stops well before that age. The plot in Figure 2, which aims to show the screening preventable potential, is thus restricted to ages 25-80 for cancers potentially being able to be prevented by screening. We have explained it in the revised method section, and in the footnote of re-drawn Figure 1.*

7. Discussion: use of the term ‘impact numbers’ needs to be revised because it’s confusing. E.g. para 2, lines 3-4, ‘A program could e.g. decide to use a screening test that does not test for HPV types with high impact numbers’ I think it means if the numbers NNS and NNF are high – but it sounds like the opposite, that the HPV type wouldn’t be included if the impact of testing for it were high.

*Reply: This is correct- a high impact number does imply a low impact and we realize that this can be confusing. We have systematically rewritten to write the more intuitive “ low impact” instead.*

8. Discussion, p8, para 3: ‘This suggests that strategies where only selected types are screened for or followed-up may be particularly appropriate in younger age groups.’ It would be good to see a bit about the ethical aspects and public communication of deciding not to screen for an oncogenic HPV type that is included in a test and for which a result would be available, even if the number needed to screen is high.

*Reply: We have included in the forth paragraph of the discussion about the ethical aspects. Specifically: The different impacts of screening for different HPV types has a several ethical aspects. For example, if it is well motivated to screen for some types but not for others, screening for all of them as a package without informing the women about the different impacts is ethically questionable.
The knowledge about the different impacts of different types could e.g. be used when selecting an HPV test with an optimal impact. If an HPV test is being used that does test for HPV types with low oncogenicity, it would seem appropriate to inform the women about the associated risk of that specific HPV types and whether the risk does warrant a follow-up or not.*

Requests from the editors:

GENERAL COMMENTS

Please respond to all editor and reviewer comments.

1. Please include page numbers and line numbers in the manuscript file. Use continuous line numbers (do not restart the numbering on each page).

*Reply: added.*

2. Please cite the reference numbers in square brackets (e.g., “We used the techniques developed by our colleagues [19] to analyze the data”). Citations should be preceding punctuation.

*Reply: fixed.*

3. Please cite your Supporting Information as outlined here: [https://eur01.safelinks.protection.outlook.com/?url=https%3A%2F%2Fjournals.plos.org%2Fplosmedicine%2Fs%2Fsupporting-information&data=05%7C01%7Cjoakim.dillner%40ki.se%7C70fb735d4b1947853e1508db5d3cff70%7Cbff7eef1cf4b4f32be3da1dda043c05d%7C0%7C0%7C638206288801488281%7CUnknown%7CTWFpbGZsb3d8eyJWIjoiMC4wLjAwMDAiLCJQIjoiV2luMzIiLCJBTiI6Ik1haWwiLCJXVCI6Mn0%3D%7C3000%7C%7C%7C&sdata=3mcfvN%2Ba0ALLHcuaeduN%2BeJmQPZ%2FIzXk7ocrMIrBSGg%3D&reserved=0](https://journals.plos.org/plosmedicine/s/supporting-information)
*Reply: fixed.*

4. Please ensure consistency in your number format and revise your manuscript accordingly including the Supplementary Material (51·4% or 51.4%; 1000 or 1,000).

*Reply: fixed.*

5. Please remove the Statements section from your main manuscript. The data should only be included in the corresponding section in the online submission form. 
*Reply: removed.*

TITLE
6. Please revise your title according to PLOS Medicine's style. Your title must be nondeclarative and not a question. It should begin with main concept if possible. "Effect of" should be used only if causality can be inferred, i.e., from an RCT. Please place the study design in the subtitle (ie, after a colon).
*Reply: Revised as instructed. We added the study design “population-based estimation” as the subtitle.*

ABSTRACT

7. Please structure your abstract using the PLOS Medicine headings (Background, Methods and Findings, Conclusions). The Methods and Findings sections should be combined into one section, “Methods and findings”."

*Reply: revised.*

8. Please define HPV at first in the abstract.
*Reply: Done.*

Abstract Background:
9. *Provide the context of why the study is important. The final sentence should clearly state the study question.

*Reply: revised.*

10. Please ensure that all numbers presented in the abstract are present and identical to numbers presented in the main manuscript text.

*Reply: ensured.*

11. PLOS Medicine requests that main results are quantified with 95% CIs as well as p values.  Please include.  When reporting p values please report as p<0.001 and where higher as the exact p value p=0.002, for example.  For the purposes of transparent data reporting, if not including the aforementioned please clearly state the reasons why not.

*Reply: 95% confidence intervals were revised to the requested format. The confidence intervals were from bootstrap resampling (gives confidence intervals but not p-values).*

12. Please include any important dependent variables that are adjusted for in the analyses.

*Reply: we added the adjustment of age, which was the main and only variable controlled for.*

13. Throughout, suggest reporting statistical information as follows to improve clarity for the reader “22% (95% CI [13%,28%]; p</=)”. Please amend throughout the abstract and main manuscript.
Please note the use of commas to separate upper and lower bounds, as opposed to hyphens as these can be confused with reporting of negative values.

*Reply: revised.*

14. Please define all abbreviations used for statistical reporting at first use.

*Reply: revised.*

15. When a p value is given, please specify the statistical test used to determine it.

*Reply: fixed.*

16. Please provide brief demographic details of the study population (e.g. sex, age, ethnicity, etc)
*Reply: The study applies only to women, has no age restrictions and all women in Sweden are eligible. In Sweden, it is for ethical reasons not allowed to register ethnicity and thus no statement about that is added.*

17. In the last sentence of the Abstract Methods and Findings section, please describe the main limitation(s) of the study's methodology.

*Reply: added.*

Abstract Conclusions:
18. *Please begin your Abstract Conclusions with "In this study, we observed ..." or similar, to summarize the main findings from your study, without overstating your conclusions. Please emphasize what is new and address the implications of your study, being careful to avoid assertions of primacy. 
*Please avoid vague statements such as ""these results have major implications for policy/clinical care"".  Mention only specific implications substantiated by the results.

*Reply: revised.*


AUTHOR SUMMARY

19. At this stage, we ask that you include a short, non-technical Author Summary of your research to make findings accessible to a wide audience that includes both scientists and non-scientists. The Author Summary should immediately follow the Abstract in your revised manuscript. This text is subject to editorial change and should be distinct from the scientific abstract. Please see our author guidelines for more information: [https://eur01.safelinks.protection.outlook.com/?url=https%3A%2F%2Fjournals.plos.org%2Fplosmedicine%2Fs%2Frevising-your-manuscript%23loc-author-summary&data=05%7C01%7Cjoakim.dillner%40ki.se%7C70fb735d4b1947853e1508db5d3cff70%7Cbff7eef1cf4b4f32be3da1dda043c05d%7C0%7C0%7C638206288801488281%7CUnknown%7CTWFpbGZsb3d8eyJWIjoiMC4wLjAwMDAiLCJQIjoiV2luMzIiLCJBTiI6Ik1haWwiLCJXVCI6Mn0%3D%7C3000%7C%7C%7C&sdata=mj4v5p0XzYUS%2BNnTvEXyH2HtuKKsPcj4wVHcZP0hixk%3D&reserved=0](https://journals.plos.org/plosmedicine/s/revising-your-manuscript#loc-author-summary).

The summary should include 2-3 single sentence, individual bullet points under each of the questions.
It may be helpful to review currently published articles for examples which can be found on our website here [https://eur01.safelinks.protection.outlook.com/?url=https%3A%2F%2Fjournals.plos.org%2Fplosmedicine%2F&data=05%7C01%7Cjoakim.dillner%40ki.se%7C70fb735d4b1947853e1508db5d3cff70%7Cbff7eef1cf4b4f32be3da1dda043c05d%7C0%7C0%7C638206288801488281%7CUnknown%7CTWFpbGZsb3d8eyJWIjoiMC4wLjAwMDAiLCJQIjoiV2luMzIiLCJBTiI6Ik1haWwiLCJXVCI6Mn0%3D%7C3000%7C%7C%7C&sdata=oo4P355HLjbgAyRYnVDxTJ0vxNsSK9AwEj48cknPRBI%3D&reserved=0](https://journals.plos.org/plosmedicine/)
*Reply: Author summary is added.*


INTRODUCTION

20. p.3: Please define WHO in the Introduction.

*Reply: revised.*

21. p.3: Please define HPV in the Introduction.

*Reply: revised.*

22. p.3: Please define IARC in the Introduction.
*Reply: revised.*

23. Please address past research and explain the need for and potential importance of your study. Indicate whether your study is novel and how you determined that. If there has been a systematic review of the evidence related to your study (or you have conducted one), please refer to and reference that review and indicate whether it supports the need for your study. 
*Reply: Introduction was largely revised. More references were added in the third paragraph including a systemtic review.*

24. Please remove the study results and/or conclusion from the Introduction.
*Reply: revised.*

25. Please conclude the Introduction with a clear description of the study question or hypothesis.
*Reply: I think in the revised text we are clear about the purpose and study aim: As specific impact had not previously been calculated, we wished to calculate the impact.*


METHODS AND RESULTS

26. Please ensure that the study is reported according to the STROBE guideline, and include the completed STROBE checklist as Supporting Information. Please add the following statement, or similar, to the Methods: "This study is reported as per the Strengthening the Reporting of Observational Studies in Epidemiology (STROBE) guideline (S1 Checklist)."
The STROBE guideline can be found here: [https://eur01.safelinks.protection.outlook.com/?url=http%3A%2F%2Fwww.equator-network.org%2Freporting-guidelines%2Fstrobe%2F&data=05%7C01%7Cjoakim.dillner%40ki.se%7C70fb735d4b1947853e1508db5d3cff70%7Cbff7eef1cf4b4f32be3da1dda043c05d%7C0%7C0%7C638206288801488281%7CUnknown%7CTWFpbGZsb3d8eyJWIjoiMC4wLjAwMDAiLCJQIjoiV2luMzIiLCJBTiI6Ik1haWwiLCJXVCI6Mn0%3D%7C3000%7C%7C%7C&sdata=Wz7lCFm8xt9nY596SwtJ253m8y7PpbxRwJJLONLylYA%3D&reserved=0](http://www.equator-network.org/reporting-guidelines/strobe/)
When completing the checklist, please use section and paragraph numbers, rather than page numbers.

*Reply: Checklist is added and mentioned in the method section.*


27. Did your study have a prospective protocol or analysis plan? Please state this (either way) early in the Methods section.
a) If a prospective analysis plan (from your funding proposal, IRB or other ethics committee submission, study protocol, or other planning document written before analyzing the data) was used in designing the study, please include the relevant prospectively written document with your revised manuscript as a Supporting Information file to be published alongside your study, and cite it in the Methods section. A legend for this file should be included at the end of your manuscript. 
b) If no such document exists, please make sure that the Methods section transparently describes when analyses were planned, and when/why any data-driven changes to analyses took place. 
c) In either case, changes in the analysis-- including those made in response to peer review comments-- should be identified as such in the Methods section of the paper, with rationale.
For all observational studies, in the manuscript text, please indicate: (1) the specific hypotheses you intended to test, (2) the analytical methods by which you planned to test them, (3) the analyses you actually performed, and (4) when reported analyses differ from those that were planned, transparent explanations for differences that affect the reliability of the study's results. If a reported analysis was performed based on an interesting but unanticipated pattern in the data, please be clear that the analysis was data-driven.¨

*Reply: The main purpose of the study was to calculate the impact of screening for different HPV types. The method used is described in reference 12 and this reference therefore corresponds to the prospective analysis plan. In addition, personally identifiable individual level data was used to obtain the input values for the calculations. The obtaining and processing of personal data was performed with specific ethical review board permissions (that contain a study plan) and these are enclosed as supplementary materials and mentioned in the method section.*

28. PLOS Medicine requests that main results are quantified with 95% CIs as well as p values.  Please include.  When reporting p values please report as p<0.001 and where higher as the exact p value p=0.002, for example.  For the purposes of transparent data reporting, if not including the aforementioned please clearly state the reasons why not.

*Reply: Estimations by bootstrap resampling gives the confidence intervals, but not p-values.*

29. Please include any important dependent variables that are adjusted for in the analyses.

*Reply: We added a paragraph in the method section for age standardization and stratification, and motivated why no more variables were adjusted.*


30. Suggest reporting statistical information as detailed above – see under ABSTRACT
*Reply: revised.*

31. p.4: Please change “This study was based on entire population of women live in Sweden from 1990s to 2019, […]” to “This study was based on the entire population of women living in Sweden from the 1990s to 2019, […]”. Please check your manuscript carefully for grammar, spelling and punctuation.
*Reply: revised.*

32. p.4: “We integrated these data sources through cross-linkage at the individual level using personal identification number, and generate fundamental parameters of […]” – please change “generate” to “generated” and ensure to keep the tense consistent throughout the manuscript.
*Reply: revised.*

33. p.4: Please define PCR in the Methods.
*Reply: revised.*

34. p.4: Please write “Table 1”/“Figure 1” instead of “table 1”/”figure 1” and check throughout your manuscript.
*Reply: revised.*

35. p.7: Please change “pseudo scenario” to “pseudo-scenario” and check carefully throughout your manuscript to keep a consistent spelling.
*Reply: revised.*

36. p.7: Please remove the comma in “Figure 4 compares the prevalence and risk profile across 14 HPV types in the pseudo scenario of no screening, in a two-dimensional graph”.
*Reply: revised.*

37. p.7: Please change “Y-axis displays the age-standardized prevalence in percentage of each type in the population, and x-axis displays the incidence of cervical cancer among women positive for each HPV type in absence of screening.” to “The y-axis displays the age-standardized prevalence in percentage of each type in the population, and the x-axis displays the incidence of cervical cancer among women positive for each HPV type in absence of screening.”.
*Reply: revised.*

38. p.7: Please re-write the sentence starting with “Screening likely to have contributed to around […]”. Editorial suggestion: Screening likely contributed to approximately 72% (69%-74%) reduction in cases caused by HPV16 and by types other than 16 and 18, whereas it contributed to only 54% (41%-63%) reduction in cases caused by HPV18 (Table 2).
*Reply: revised.*

39. p.7: “The impact numbers for cervical screening in the population, i.e NNS and NNF to detect or
prevent one case of cervical cancer, […]” – please change “i.e” to “i.e.”.
Reply: revised.

40. p.7: Please check the grammar and punctuation for the following sentence: “For HPV51 NNS and NNF could not be estimated because too few HPV51-positive cervical cancer cases were detected during 10 years in the country of Sweden) (Table 3, Figure 5, Supplementary Table 1).”
*Reply: revised.*

41. Please present numerators and denominators for percentages, at least in the Tables [not necessarily each time they're mentioned].
*Reply: revised.*


DISCUSSION

42. Please present and organize the Discussion as follows: a short, clear summary of the article's findings; what the study adds to existing research and where and why the results may differ from previous research; strengths and limitations of the study; implications and next steps for research, clinical practice, and/or public policy; one-paragraph conclusion.
Please remove all subheadings within your Discussion e.g., limitations and other considerations.

*Reply: discussion is re-organized to fit the structure.*

43. p.8: “In particular, the lower oncogenic types HPV35, 39, 51, 56, 59, 66, 68 may be considered not screen for or not directly refer to follow-up in women below age 30.” – please change to “In particular, the lower oncogenic types HPV35, 39, 51,56, 59, 66, 68 may not be considered for screening or direct follow-up in women under 30 years of age.”.

*Reply: we changed it to be: “In particular, consideration of no screening or no follow-up in women under 30 years of age may be warranted for the lower oncogenic types (HPV35, 39, 51,56, 59, 66, 68)”.*

44. p.8: Please define CIN2+ and CIN3+ in the Discussion.
*Reply: revised.*

45. p.9: The third paragraph (In general, the presented impact numbers should largely be […]) seems redundant as it repeats the statements of the previous paragraphs. Please be careful to make key statements and avoid repetition.
*Reply: Agree. We have deleted it.*

46. p.9: “Nevertheless, as these types caused very few cases regardless of screening historyimply that they are potentially negligible types to screen for.” – please change to

“Nevertheless, since these types caused very few cases, regardless of screening history, they are potentially negligible types to screen for.”.

*Reply: revised.*

FIGURES

For all Figures, please ensure that you have complied with our figures requirements [https://eur01.safelinks.protection.outlook.com/?url=http%3A%2F%2Fjournals.plos.org%2Fplosmedicine%2Fs%2Ffigures&data=05%7C01%7Cjoakim.dillner%40ki.se%7C70fb735d4b1947853e1508db5d3cff70%7Cbff7eef1cf4b4f32be3da1dda043c05d%7C0%7C0%7C638206288801800713%7CUnknown%7CTWFpbGZsb3d8eyJWIjoiMC4wLjAwMDAiLCJQIjoiV2luMzIiLCJBTiI6Ik1haWwiLCJXVCI6Mn0%3D%7C3000%7C%7C%7C&sdata=8LAsvFSuyHlQHQomExhpIcZ2y19dK4Whm05irXBAy7Q%3D&reserved=0](http://journals.plos.org/plosmedicine/s/figures).
*Reply: We need technical help to convert the figures from powerpoint to tiff or eps. Could you guide us how to do it?*

Please provide titles and legends for all figures (including those in Supporting Information files).
*Reply: added.*

Please define abbreviations used in the table/figure legend of each figure and/or table (including those in Supporting Information files).
*Reply: fixed.*

In Figure 2, please provide 95% CIs.

*Reply: added.*

Figure 3: Please define the scale on the y-axis as “%” is not sufficient in this case.

*Reply: revised.*

Figure 4: The description of the different scales used in Figure 4 (left graph x-axis on regular scale, right graph x-axis on log scale) should not be in brackets in the title, but part of the figure description.
*Reply: revised.*

Please consider avoiding the use of red and green in order to make your figure more accessible to those with colour blindness.

*Reply: we have used line patterns and text to distinguish items in addition to colors.*

Please indicate in the figure caption the meaning of the whiskers in Figure 5 and 6.
*Reply: They mean confidence intervals, which was mentioned in the figure captions.*


REFERENCES

Please ensure that journal name abbreviations match those found in the National Center for Biotechnology Information (NCBI) databases ([https://eur01.safelinks.protection.outlook.com/?url=http%3A%2F%2Fwww.ncbi.nlm.nih.gov%2Fnlmcatalog%2Fjournals&data=05%7C01%7Cjoakim.dillner%40ki.se%7C70fb735d4b1947853e1508db5d3cff70%7Cbff7eef1cf4b4f32be3da1dda043c05d%7C0%7C0%7C638206288801800713%7CUnknown%7CTWFpbGZsb3d8eyJWIjoiMC4wLjAwMDAiLCJQIjoiV2luMzIiLCJBTiI6Ik1haWwiLCJXVCI6Mn0%3D%7C3000%7C%7C%7C&sdata=rPJqp6aBiorT2%2BVbIKvpypUkUqKFGFkBwFbhA9oGTY8%3D&reserved=0](http://www.ncbi.nlm.nih.gov/nlmcatalog/journals)), and are appropriately formatted and capitalised.

Please also see [https://eur01.safelinks.protection.outlook.com/?url=https%3A%2F%2Fjournals.plos.org%2Fplosmedicine%2Fs%2Fsubmission-guidelines%23loc-references&data=05%7C01%7Cjoakim.dillner%40ki.se%7C70fb735d4b1947853e1508db5d3cff70%7Cbff7eef1cf4b4f32be3da1dda043c05d%7C0%7C0%7C638206288801800713%7CUnknown%7CTWFpbGZsb3d8eyJWIjoiMC4wLjAwMDAiLCJQIjoiV2luMzIiLCJBTiI6Ik1haWwiLCJXVCI6Mn0%3D%7C3000%7C%7C%7C&sdata=yfYe6zCWNn6%2FxodMzg4b%2BqHXtrKmO6yJyIP2TbarCOM%3D&reserved=0](https://journals.plos.org/plosmedicine/s/submission-guidelines#loc-references) for further details on reference formatting. 

Where website addresses are cited, please specify the date of access. 

*Reply: references are checked format is revised.*

Reviewer #1:

The manuscript contains the calculations for relevant impact numbers of cervical cancer screening, referred to the Swedish population. Data on HPV type-specific prevalence (in cancer cases and in population), oncogenicity and screening effectiveness were collected from different groups and over more than 20 years. By incorporating these data in the analyses, the authors have calculated the number need to screen (NNS) and the number need to follow-up (NNF) to detect or to prevent a case of cervical cancer by each HPV type. These impact numbers were substantially different among the 14 HPV types evaluated (12 high-risk and 2 probably/possibly oncogenic). On the basis of the results, the authors provide a stratification of these HPV types (as high, intermediate and lower oncogenicity), and indicate (also by age-groups) the most effective screening strategy, that can imply no screening or no follow up for infections by the lower oncogenic types, especially among the youngest age groups. The approach allows recalculation of the impact numbers over time, and this is an important aspect. The cervical cancer screening is being implemented in many countries with high-riskHPV testing as primary test; moreover, the use of a risk-based approach to manage the women with a positive screening result (where HPV genotyping could play a role as triage test) and the integration of primary (anti-HPV vaccination) and secondary (screening) preventive measures need to implement the protocols and monitor the effectiveness of the screening. The authors claim that these results could apply to other countries with similar type-specific HPV prevalences and similar screening efficiency; generalizability will probably be limited by differences in the prevalence of different HPV types (observed also within the same country) and by the substantial differences in anti-HPV vaccination coverages. Anyhow, the topic of this paper is of great importance in the field of
cervical cancer prevention, where instruments to device the best strategies to adapt and personalize the screening strategies is highly needed. Moreover, it is important to the health community at large because these changes to be effective need that all the healthcare professionals give updated and concordant informations to the women.

*Reply: Thank you for acknowledging our work. We have discussed how the impact numbers could be recalculated in other settings with different HPV type-specific prevalences, and that it can be monitored in the upcoming change of HPV type-specific prevalences.*

MINOR OBSERVATION:
-TABLE 1: a footnote reporting the distribution of the HPV types in the three subgroups could be usefully added

*Reply: The overal distribution of intermediate and lower oncogenic HPV types by screening status can be found in Table 3, if it is what is asked for. We added the footnotes of exact types included in the intermediate and lower oncogenic types.*

# Reviewer #2:

The paper represtnts a clever use of existing data. It will change our way to think about genotyping in cervical cancer screening.

*Reply:* *Thank you for acknowledging our work.*

I have only few comments to improve the roprting and a comment on the interpretation that could be a matter for discussioon. 
 
Abstract
1. I do not understand why NNS and NNF are reported only for women below 30, while for the all ages is reported only the variation without any absolute number. For the reader is not easy to follow the results as reported in the abstract.

*Reply: We added the statements for all ages in the abstract. However, we would like to keep the emphasize for young women, as it is in particular the results among the young women that have an immediate public health implication. In the author summary we stated about the implication for both young and older aged groups.*

2. I think it should be clear that protection estimates come from screening with Pap test, not HPV test. Instead of the generic disclaim "Although reasonable limits for impact numbers may differ between settings" a more specific comment on the different level of protection given by HPV now could be appropriate.

*Reply: In the abstract, we emphasized the historical context of screening attendance (Historical screening attendance was associated with a 72% reduction of cervical cancer incidence caused by HPV16…*)*, we added the descriptions on the historical screening strategies to the methods section and also added a comment on the potential protection improvement from HPV-based screening in the discussion section.*


Introduction
3. Clear and well written. I only suggest better explaining how the number need to detect and number need to prevent interacts: the two desirable effect of screening, i.e. early detection of cancers and prevention of cancers, are additive and there is no way that any positive event is counted twice.

*Reply: It is true that they were not counted twice. The “detected” cases are the cases not being prevented through screening even though screening is performed as it should have done. We added a description of the relationship between prevention and detection in the introduction.*

4. I have a comment about the type specific NNS. It can be high for two reasons, low prevalence in the population or low oncogenic risk. If the issue is low prevalence, the type specific NNS to screening is not relevant for the decision making of including it or not, since the real NNS is that for all types. The NNS of the overall screening test is not the average of the NNS of single HPV types. As a paradox if we use a taxonomy that splits the HPV 16 in different variants, also the NNS of each HPV 16 variant will increase dramatically, without changing nothing about the efficiency or effectiveness of the test.

*Reply: When designing a screening program, it is essential to know if the condition screened for is rare or common. Which conclusion to make is up to the program – we are simply providing data that can be considered.*

Methods
5. According to my understanding of the computations made by the authors, the underlying model is simple but robust; its simplicity is a strength because it is also transparent. Nevertheless, simplicity of the model implies many assumptions. I suggest to add a paragraph or a box reporting all the implicit assumptions made by your model. The main issues are two, from first glimpse of the described methods:

*Reply: The study has 3 assumptions which were previously discussed in the supplementary material. We now moved them the to the main text in the method section under each relevant paragraph: HPV type-specific prevalences in the population, calender priod stability, and cervical cancer risk between screened and unscreened population.*

6.  the model assumes a steady state, i.e. the analysis of HPV types in screened and unscreened population assumes that these proportion are the product of a stable dynamic between infections and progression to cancers in the two populations (this assumption is acceptable but should be explicit);

*Reply: This is indeed an assumption. We are happy that the reviewer thought that this was acceptable. We have also added the following clarifications in the method section about this point:*

*Since the screening coverage is above 80% in Sweden, the HPV-type-specific prevalence in the population as a whole is dominated by the prevalence in the screened population. The cervical cancer risk in the population, it is dominated by the strong effect of screening. We have in our previous study compared the cervical cancer risk between screened and unscreened group adjusting for country of birth and level of education (Wang et al., IJC 2020, reference 26), and found that these two factors did not confound the effect of screening itself. This supports that the risk in the screened population would have been the same as the unscreened population should they be unscreened.*

7. probably you are also assuming that incubation time is equal for all types. The fact that this assumption is not true is probably the explanation for a different ratio between HPV16 and HPV low oncogenic NNS and NNF across ages: the incubation time is different, so there are few cancers from low-oncogenic types in younger women.

*Reply: The calculations do* ***not*** *assume anything about incubation time et c, they are a simple number estimating the impact of screening in that age group.*

*Different incubation times by HPV type is indeed a reasonable explanation for our finding of very low impact of screening for low oncogenicity HPV types among young women. We do not need to assume the incubation time being equal across the types. If a type has long incubation time this implies a low likelihood that cancer is present now or that it would cause cancer in the upcoming 10 years.*

*The different incubation time across HPV types imply that for viruses with long incubation time, less intense screening is warranted to avoid negative outcomes of screening. This is consistent with the results of our impact estimations.*

8.      How do you treat the large part of cancers that are not positive for any HPV oncogenic type? According to table 1 and 2, it seems that they are distributed in the proportions of 16, 18 and other oncogenic but maybe I am wrong. It would be better to make an explicit statement on this point.

*Reply: In Table 1 and Table 2, they were in the denominator. It is noted in legend of Table 1 and in Table 2 now.*

9. I do not understand why all the measures are "age standardized". Actually all the relevant parameters are age-specific and all the counterfactual scenarios are estimated on a "standard" population, I do not see the need for standardizing. Particularly for type-specific HPV prevalence, standardization may be relevant only for the comparison of the screened and non-screened population that have different age structures. If this is the rationale, I suggest to explain it. All the other parameters are always age specific.

*Reply: Age standardization was performed to i) control for the different age distribution in the screened and unscreened population in general, as you pointed out, and ii) to report the overall impact numbers not limiting to populations with the same age structure. We have added motivation for age-standardization in the revised method section.*


Results
10. First line of the results and figure 2 reports incidence in 2004-2011, while table two reports cancers from 2002 to 2011. Can you clarify?

*Reply: we clarified it in the legend of revised figure 1.*

11. Please define the time of incidence is per 100,000/y or cumulative for the 8-year period?

*Reply: it is per 100,000 person-years for each year, average for 8 years. We clarified it in the text and figure.*

12. Table 2. Could you report also the protection of screening for HPV-negative cancers?

*Reply: we added it in table 2 now. Regarding this, we think it is an aditional limitation: the negativivity of oncogenic HPV is from FFPE tumor blocks, and we know little about their HPV infection status and types in the years before cervical cancer diagnosis, thus we cannot predict how HPV-based screening would impact this group. We will be able to know more in the near future with accumulated data from HPV-based screening. Nonetheless, since few cervical cancer cases in young women were negative for oncogenic HPVs in tumor blocks, our estimates for young women were barely affected by this issue. We added this as a limitation in the discussion section.*

13. Figure 2. y-axis report if the incidence is annual or cumulative.
*Reply: added.*

Discussion 
14. pag 8 second paragraph, I do not agree with the sentence: "An alternative strategy to use the NNS data could e.g. be to decide that detection of HPV types with high impact numbers is a normal screening result". As discussed before, if we adopt a different taxonomy and we give a different name to all HPV16 variants, the NNS will increase for each of these, the same for HPV 45 in some populations where it is extremely rare, but it will be always worth to include it among the oncogenic ones. Furthermore, low prevalence of a HPV type increases the type specific NNS but not the burden of assessment tests. NNS is relevant to decide if we have to screen or not at all, but it is not relevant for deciding which type should be included in the set of HPV types to test.

*Reply: We agree that this sentence could easily be misinterpreted so we have removed it.*

15. pag 9, second paragraph: when mentioning the limitation that the screening effectiveness assessment was conducted for cytology-based screening, I think it is worth to report how much is expected to improve the protection with HPV based test, particularly for adenocarcinoma; an estimate could be given based on the pooled analysis of European trials (reference n 1) .

*Reply: we agree and added it in the discussion.*

# Reviewer #3:

Alex McConnachie, Statistical Review

This review considers the statistical elements of the paper by Wang et al, looking at impact numbers in cervical screening in Sweden, in relation to HPV type and age.

The paper is a very nice analysis of routinely collected data, and data collected as part of other studies, and an excellent demonstration of how this can be used to inform public health policy. The impact numbers and how they are calculated are explained in great detail. The use of bootstrapping to generate confidence intervals is appropriate. The presentation is, on the whole, very clear.

I have one or two comments, which are minor, bordering on the trivial.

1. The date range for the study population is a little vague; should exact dates be given?

*Reply: we now explained the study periods more clearly in the legend of revised figure 1.*

2. The paragraph giving descriptions of the NNS and NNF to detect or prevent one case is very good, in that it accurately describes each parameter. However, it is quite dense. Could some consideration be given to presenting these as formulas? Would some readers find that easier to follow?

*Reply: agree. Formulas are added.*

3. In Table 2, do the columns headed "Population" add anything? Could it be made clearer that the columns headed "Screened in last 10 years" actually relate to the hypothetical situation where everyone is screened (and similarly for the "Unscreened in last 10 years")? It is clear from the footnotes, but could perhaps be clearer in the headers.

*Reply: agree. Have removed it.*

4. In Table 4, the columns are labelled as "Number needed to screen to detect (or to prevent) one case". However, only the top half of the table reports NNS, and the bottom half gives NNF. Maybe the columns should simply say "To detect one case" and "To prevent one case".

*Reply: thank you for carefully pointing it out. We revised it.*

5. In Figure 2, the abbreviation "ASR" is not defined.

*Reply: we added the explanation.*

6. In Figures 5 and 6, the confidence intervals are asymmetrical, and those to the left of each panel are quite compressed. Would the figures work better with the x-axes on a log scale? Each figure has a different range on the x-axis - would showing on a log scale allow these to have a common scale? Would that be slightly better?
*Reply: We tried to make the figures in a panel with the common scale on the log scale, as below. The conficence intervals remained asymmetrical because they are not from model but from bootstrap resampling. However we found this transformation does not really meet our purpose for the graph presentation, as it somehow visually reduces the substantial difference of the impact numbers across HPV types. We suggest to keep the linear scale, as is.*
.

# Reviewer #4:

1. The current revision of the paper includes an interpretation in the abstract that is non-committal. Focusing on the NSN and NNF for women over 30 years might be more useful for an interpretation to put money toward screening and following up the abnormals.

*Reply: We have added the statements for all ages in the abstract. However, we would like to keep the emphasize for young women, as it is in particular the results among the young women that have an immediate public health implication.*

2. Methods - it is inappropriate to classify HPV types by whether or not they are in the Merck based nonovalent vaccine- do not let industry drive your definitions, let the science. Why don't you group the HPV types in the way that BD has grouped them according to aggressiveness of oncogenesis with 16/18/31 as separate highest risk types, and then 33/58 together, then 52 and 45 separately as medium high risk, then  type 51 alone as a lower risk HR type, then 35/39/68 grouped together, and 56/59/55 grouped together.  At least this grouping of HPV types is associated with risk of CIN 3+ over time.

*Reply: Using a specific HPV test (BD) to define classifications of HPV types would be to let industry drive the definitions. The current classification is based on the ranking of WHO/IARC (which was the evidence that the HPV vaccine was based on). We deleted the statement about vaccination and cited the evidence from WHO/IARC.*

3. What is your assumption about HPV vaccination? You are using the Swedish population and creating pseudo scenarios that are dependent on the current HPV vaccination type and uptake remaining constant. Is this true?  How does this limit your conclusions?

*Reply: the current data did not include birth cohorts of women being vaccinated in school-based or similar high-coverage vaccination programs. We clarified this in the revised method section. And we have added discussion on the possibility and necessity of monitoring the impact numbers along with the expected HPV type-specific prevalence reduction after HPV vaccination, which will be necessary to inform the screening program for continuously improving its impact.*

4. Your definition of NNS and NNT are too simplistic.  You are creating an ideal situation where you only value screening for follow up if a cancer is detected.  Screening is always a BALANCE between over-screening and leaving cancers on the table.  You might check with a Bayseian mathetmatician about the pseudo scenarios you have created that the level of error inherent in each.

*Reply: We have calculated* *both the impact in terms of cancer prevention and of cancer detection. We now made it more clear in the revised introduction section.*

5. Figur2-What percentage of the population did not have a screen prior to her diagnosis? Why was she diagnosed with cervical cancer? symptoms? incidental for something else? what is the age distribution of these cancers?

*Reply: The percentage of the population that is uncreened is about 10%-20% in Sweden. It is now mentioned in the revised method section, and also mentioned in S1 appendix. Figure 2 aims to show the cancer prevention effect in the population. The cancer cases that were “screened” was defined as having a test in the past 10-0.5 years prior to cancer diagnosis, which is the period potentially to prevent the cancer. Tests within half a year prior to cancer diagnosis were part of the cancer diagnosis. We made it clear in the revised method section. With the same definition, percentage of the cases with and without screening in the preceding 10-0.5 years by age can be referred from Table 1.*

6. Figure 4 is not offering any information other than a plot.

*Reply: Together with the table for screening prevention potential by HPV-type, they present in a pedagogic and clear manner the key three elements underlying the impact number calculation: type-specific prevalence, oncogenicity and screening effectiveness. We agree that it did not add any new information for the field, so we moved it to the supplementary material.*

7. Age stratification needs to be above 30, not above 50 - or you need to provide adequate justification for not using 30 and older for your analysis.

*Reply: The screening interval has been different for women below or above 50. It is now explained in S1 appendix.*

8. Discussion section needs to state that the limitation of racial/ethnicity diversity in Sweden means these data cannot be extrapolated to other continents where the population homogeneity is dissimilar.

*Reply: We have no data on race/ethnicity as it is considered unethical to register that in Sweden. The potential variation across racial/ethnic groups may result in differences in HPV-type-specific prevalences and screening participation. We mentioned in the discussion that the impact numbers can readily be measured in other populations with other HPV-type-specific prevalences. The impact number calculation was under the pseudo-scenarios if all women are screened and if all are unscreened. The exact screening participation is thus not necessary to consider when designing which HPV types should be screened and followed.*

9. HPV 18 discussion is interesting - was the screening cytology based? if we went to HPV primary testing would we have missed those cancers?  if we went with HPV 18 testing followed by Dual Stain, would those cancers have been missed in screening?  Is it a fault of the screening that these cancers were not detected, or was it a fault of the women not coming in for screening?

*Reply: The result shows that the protection of the screening was lower for HPV18. We have inserted a discussion that maybe specific screening for HPV18 could improve on this. We also added in the discussion about how much the HPV-based screening may improve the prevention for HPV18 related cervical cancer. This problem is not a fault of the women not coming in for screening, as the conclusion is based on the screening effectiveness comparison across varied HPV types, all among women participation in screening.*
