# Supplementary material for: Impact of cervical screening by human papillomavirus genotype: Population-based estimations
Source: PLoS Med. 2023 Oct 27;20(10):e1004304. doi: 10.1371/journal.pmed.1004304 (PMC10637721; doi:10.1371/journal.pmed.1004304)
Supplement: Attachment — Submitted filename: RespondsToComments_JW20230912.docx [file pmed.1004304.s007.docx]

Responses to Editors’ and Reviews’ comments

Requests from Editors:

GENERAL

Thank you for considered and detailed responses to editor and reviewer comments.

Please see below for further minor points that we request you respond to in full.

Please add 'years’ to ages stated throughout your manuscript.

Reply: added.

We note that the Supporting Information file S2 (Figure S2) provides important details for the understanding of the paper. We leave it to your discretion whether you wish to include it in the main paper.
Reply: we agree, and have now moved it in main paper. The order of figures are revised accordingly.

Thank you for providing a copy of your study protocol (S7) as a supporting information file. Please send this in the original language and an English translation. Please detail any deviations from this study protocol in the Methods section of your manuscript.

Reply: translation of S7 (now S4 after re-order) is added.

ACADEMIC EDITOR COMMENTS

Thank you for the opportunity to read the revised manuscript. The authors have made extensive changes and the description is much clearer. I apologise for the rather impolite comment about the writing in the previous version. The authors had addressed my main comments.

Reply: thank you. We appreciated the criticism, and the revision did really improve the clarity.

1. Introduction: line 83, “Impact numbers [12] are a suitable measurement.” Whilst these are described in the next para, it would help to at least introduce ‘population’ and ‘disease’ impact numbers because they are not widely used terms.

Reply: a brief description of population and disease impact number is added.

2. Line 144: spelling, ”assumPtions”

Reply: changed

3. Line 155: wording, suggest “We found two publications suggesting…”

Reply: changed

4. Line 177: “HPV vaccination has yet noticeably affected the study population…” should it be “…has NOT yet…”?

Reply: Correct. We have added “not”.

5. Fig 1: I cannot see where footnote c refers to

Reply: the footnote orders are now revised.

6. Lines 370-1: “NNS will therefore be constantly monitored…to timely adjust screening strategies.” This implies that a decision to choose which HPV types should be followed up has already been made. However, in response to Reviewer #2’s comment about HPV type-specific prevalence, the authors say, “Which conclusion to make is up to the program – we are simply providing data that can be considered.” Can the authors clarify whether this strategy has already been adopted?

Reply: no, it has not been adopted. To avoid confusion, we revised the sentence in the discussion to “NNS will therefore need to be constantly monitered along with the population HPV vaccine coverage in each age group, in order to obtain timely data on impact by HPV type.”

7. Line 411: wording “…to timely inform the screening program for adjustment.” As above, this sentence needs to be clarified – and made grammatically correct.
Reply: similar to above, we revised the sentence to “Impact numbers can be monitored over time, following the change of HPV type-specific prevalence and screening effectiveness, to timely provide data to the screening program for consideration of possible adjustment.”

EDITORIAL COMMENTS

When revising your manuscript, please consider that your manuscript needs to be accessible to a wide audience and aim to improve your writing for this purpose.

Reply: Editors and reviewers’ comments in this second-round review has helped sufficiently on improving the accessibility by audience. We are grateful and have changed them all accordingly.

COMPETING INTEREST

You indicated that authors KS has received research grants from Merck and Co, LLC. For authors with ties to industry, please indicate whether any of the interests has a financial stake in the results of the current study.

Reply: this interest has no financial stake in the result of the current study.

DATA AVAILABILITY STATEMENT

Thank you for agreeing to make your data available. At this time, please provide the link to the data repository and accession numbers required for access.
Reply: it is now provided:

https://doi.org/10.23728/b2share.ffe80b8d159441a7b818070cb8cc5482

http://hdl.handle.net/11304/60c49144-6d1e-4d18-949d-2af7a48ce0c8

TITLE

Since the title must be nondeclarative, we suggest changing your title to “Outcomes of cervical screening by human papillomavirus genotype: a population-based study in Sweden” or similar. We suggest changing the short title to “HPV type-specific outcomes in cervical screening” or similar.
Reply: we deem that the term “impact” is better than “outcome” for this paper, so we revised the title to “Impact of cervical screening by Human Papillomavirus genotype: Population-based estimations”. And the short title to “HPV type-specific impact in cervical screening”. Are they still declarative?

ABSTRACT

l.18 suggest: “Cervical screening programs use testing for human papillomavirus (HPV) genotypes.”

Reply: changed.

l.32: Please change ‘can’ to ‘could’.
Reply: changed.

In the last sentence of the Abstract Methods and Findings section, please describe the main
limitation(s) of the study's methodology. The statement “However, it can readily be recalculated in other settings and monitored when HPV-type specific prevalences change.” should be included in the discussion (We suggest changing the statement to ““However, it can readily be recalculated in other settings and monitored when HPV-type specific prevalence changes.”).

Reply: revised.

Please revise your Abstract Conclusion. The statement that you found different screening impacts for different HPV types is very vague. Also, findings observed in your study should be presented in past tense. We suggest, “In this study, we observed that the impact of cervical cancer screening varies depending on the HPV type screened for. Estimating and monitoring the impact of screening by HPV type can facilitate the design of effective and efficient HPV-based cervical screening programs.” or similar.

Reply: we agree, and have accepted your suggestion.

AUTHOR SUMMARY

Your author summary requires revision. The summary should include 2-3 single sentence, individual bullet points under each of the questions (Why Was This Study Done? What Did the Researchers Do and Find? What Do These Findings Mean?). Please note that the text should be distinct from the scientific abstract. In the final bullet point of ‘What Do These Findings Mean?’, please describe the main limitations of the study in non-technical language.

It may be helpful to review currently published articles for examples which can be found on our website here <https://journals.plos.org/plosmedicine/>
Reply: author summary is re-written.

INTRODUCTION

l.62: Please define ‘HPV’ at first use (you first define ‘HPV’ in l.64).

Reply: revised.

l.77: Please change ‘suggest’ to ‘suggests’.
Reply: revised.

l.102: Please exchange ‘hope’ with ‘aim’.

Reply: revised.


METHODS AND RESULTS

Please ensure that, when possible, the 95% confidence intervals quantified for the main results are reported in the main text.

Reply: we have now added the 95% confidence intervals of the impact numbers in the result section.

For description of age, please add ‘years’ (e.g. l.133 ‘women aged 23-29’). Please revise throughout the entire manuscript.
Reply: added.

I.121 onwards: would benefit from being split into shorter paragraphs to improve reader accessibility.

Reply: it is split into two paragraphs, with the assumptions being the second paragraph.

l.122: Please change to “women in the capital region of Stockholm”.

Reply: changed.

l.144: Please change ‘assumtions’ to ‘assumptions’.

Reply: changed.

l.155: Please change to “We found two publications suggesting […]”.
Reply: changed.

l.163: Please change the format of ‘4254’ to ‘4,254’. Please ensure consistency in number format and revise throughout the entire manuscript (e.g. l.166 ‘2850’ or l.206 ‘1000’).
Reply: changed throughout.

l.177-179: Please clarify whether HPV vaccination has or has not yet affected the study population. Editorial suggestion: “HPV vaccination has not yet had a noticeable impact on the study population included in this study: data were not available from birth cohorts of women vaccinated in the school-based or similar high-coverage HPV vaccination program.”

Reply: exactly as the editor suggested, we added “not” before “yet”.

l.184: We note that you usually write the word “other” in quotation marks (here: other 12 types). Please revise and ensure to use a consistent format throughout the entire manuscript.

Reply: quotation mark is added at this place, and we checked that all other “other”s for the 12 types are in quotation marks.

l.211-212: Please note that the citation does not meet the requirement of PLOS reference formatting. Please remove ‘Heller et. al'.

Reply: removed.

I.252: This statement should be presented early in the method section. Please move to perhaps follow the subheading at line 105.

Reply: moved. Orders of support materials are changed accordingly.

I.274: It might be helpful to re-define the population size such that ‘30%’ can be quantified.

Reply: yes. Population size is now added.

l.279: Please change ‘S2 Figure’ to ‘Figure S2’.

Reply: Changed.

l.286: Please add a comma following ‘and 33’.

Reply: added.

ll.283-287: Please note that results should be described in past tense.
Reply: changed to past tense.

I.302/305: Suggest ‘lower’ instead of ‘reduced’.

Reply: changed.

DISCUSSION

l.337: Please change ‘has’ to ‘have’.

Reply: changed.

l.341: Please change ‘does test’ to ‘tests’.

Reply: changed.

l.386: Please change ‘Data from European HPV-based screening trial’ to ‘Data from a European HPV-based screening trial’.
Reply: changed.

l.391: Please change ‘limitation of study’ to ‘limitation of the study’.
Reply: changed.

l.392: Please change ‘method’ to ‘methods’.
Reply: changed.

l.404: ‘Possibly/probably’, please use one or the other of the two words but not both.

Reply: changed.


FIGURES

Please consider avoiding the use of red and green in order to make your figure more accessible to those with colour blindness.

Reply: red and green in figure 2 and figure 3 are revised. We also changed the legend, so it now uses line pattern to distinguish groups other than color.

Figure 1: Please cite the reference numbers in square brackets as done in the main text. Citations should be preceding punctuation.

Reply: cited.

Figure 1: Please check the footnotes. The figure does not contain footnote ‘c’, but contains an asterisk which is not defined. Please define ‘HPV’, ‘FFPE’. For description of age, please add the unit ‘years’.

Reply: footnote orders are checked and revised. Asterisks are changed to be in footnotes. HPV and FFPE are defined. “Years” are added for ages.

Figure 2: Please add a unit for ‘age’.

Reply: added.

Figure 3: Please add a unit for ‘age’. Please define ‘HPV’.

Reply: unit of age is added in graph. HPV is defined in title.

Figure 4: Please define ‘HPV’. Please clearly define the meaning of the dots and lines for the reader. Perhaps, ‘Impact numbers (dots) and 95% confidence intervals (lines)…’ in the title?

Reply: HPV is defined in title, and “dots” and “lines” are added in title as suggested.

Figure 5: Please add a unit for ‘age’. Please define ‘HPV’. Please add a reference for the definition of the oncogenic types (as done in Table 1). Please clearly define the meaning of the dots and lines for the reader. Perhaps, ‘Impact numbers (dots) and 95% confidence intervals (lines)…’ in the title?

Reply: age group (in years) is added as y-axis label. HPV is defined in title. Reference is added in footnotes. “Dots” and “lines” are added in title as suggested.

TABLES

Table 1: Please add a unit for ‘age’. Please define ‘HPV’. Please cite the reference numbers in square brackets.

Reply: unit of age is added. HPV is defined. Reference number is added.

Table 2: Please revise the number format (4254 versus 4,254). Please change ‘pseudo scenario’ to ‘pseudo-scenario’. Please add a unit for ‘age’. Please define ‘HPV’. In the footnote ‘e’, please write ‘age-standardized’ with a capital ‘A’. Where is footnote ‘b’ in the table?

Reply: number format is changed. “Pseudo scenario” has changed to ‘pseudo-scenario’. Unit of age is added. HPV is defined in the title. “age-standardized” is changed to “Age-standardized”. Figures for footnote b was deleted in last revision. The order of footnotes is now changed.

Table 2: The definition of footnote ‘f’ might be misleading. We suggest: “Difference between the estimated number of cases in pseudo-scenario that all women were unscreened in last 10 years and the estimated number of cases in pseudo-scenario that all women were screened in last 10 years”.
Table 2: In footnote ‘g’, please introduce ‘CI’ as the abbreviation for ‘confidence intervals’.

Reply: f and g are revised accordingly.

Table 3: For clarity, we suggest exchanging the asterisk (*) and the slash (/) with ‘multiplied by’ and ‘divided by’. Please revise the number format (4254 versus 4,254). Please define ‘HPV’. Please add a unit for ‘age’. Please define ‘+∞’.

Reply: * and / are changed. Number format is changed. HPV is defined in the title. Unit of age is added. ∞ is defined in footnote.

Table 4: Please revise the number format (4254 versus 4,254). Please define ‘HPV’. Please add a unit for ‘age’. Please add a reference for the definition of the oncogenic types (as done in Table 1).

Reply: Number format is changed. HPV is defined in the title. Unit of age is added. Reference is added.

SUPPLEMENTARY MATERIAL

S1 Appendix: Please add a unit for ‘age’. Please define ‘HPV’ at first use. Please replace the word ‘cited’ with ‘Accessed’. Is the 1st ref a web ref? If so, please include the access date.

Reply: HPV is defined in the title. Unit of age is added. Cited is changed with “Accessed”. Access date is added for the 1^st^ ref.

S3 Table: Please define ‘CL’ and the meaning of ‘+∞’. Please revise the number format (4254 versus 4,254).

Reply: CL and ∞ are defined. Number format is changed through adding 1000 separator “,” to all number.

S3 Table A: Please add a definition for ‘all ages’. Could the CIs be presented in one column and separated by a comma?

Reply: Specific age range is added. CIs are presented in one column, but separated by “-“ to avoid confusion with 1000 separator.

S3 Table B: Please add a unit for ‘age’. Please add a reference for the definition of the oncogenic types (as done in Table 1).

Reply: unit of age is added. Reference is added.

REFERENCES

Please revise reference 33 regarding the abbreviated journal title. Please ensure that journal name abbreviations match those found in the National Center for Biotechnology Information (NCBI) databases (<http://www.ncbi.nlm.nih.gov/nlmcatalog/journals>) and are appropriately formatted and capitalised.

Reply: changed.

SOCIAL MEDIA

To help us extend the reach of your research, please provide any Twitter handle(s) that would be appropriate to tag, including your own, your coauthors’, your institution, funder, or lab. Please respond to this email with any handles you wish to be included when we tweet this paper.
Reply: please tweet @DillnerJoakim


Comments from Reviewers:

Reviewer #1: The authors have adequately responded to my comment, and I have no additional observations.

Reply: thank you very much.


Reviewer #2: The authors improved the manuscript that is much clearer now.
There is still an issue that I cannot agree and that should be discussed at least: the interpretation of the type-specific NNS.
The authors answered to my comment #4 as follows: "When designing a screening program, it is essential to know if the condition screened for is rare or common. Which conclusion to make is up to the program - we are simply providing data that can be considered." I do not think this sentence applies to single genotypes. In fact, we start from HPV16 that is the most common and the most oncogenic type, then the decision, for the first level test of screening, is if we want to screen for HPV16 alone or HPV16 + the second most oncogenic type (let's say HPV18), therefore the condition HPV16+HPV18 will be more common than HPV16 alone, and so on. Single genotypes with high NNS will never be rare as condition to be screened, because they will be always considered as add on to a pool of other genotypes. Therefore, the NNF will determine if they have enough risk to be included in an efficient screening. NNS may give an idea of the public health impact of including a genotype or not, but cannot help to make the most efficient selection of types.
In the discussion, I suggest to revise the sentence "If the NNS for a particular HPV type is high, meaning that the impact of the type to the screening program is low, consideration could be given as to whether screening could be discontinued for that type" (lines 318-320). This is surely true for a high NNF, but for a NNS the considerations are different: a high NNS may give a low value if we have to choice between a commercial test including that type and another not including it, but this is a different situation from deciding to dismiss screening for a single type.

Reply: We agree that the situation when discontinuing ongoing screening is more complicated compared to deciding to not include new screening.  We have changed the sentence to “If the NNS for a particular HPV type is high, meaning that the impact of the type to the screening program is low, the program may consider evaluating whether that type needs to be included in the screening.”


Reviewer #3: Alex McConnachie, Statistical Review

The authors have addressed all of my (minor) comments, and I have nothing else to add.

Reply: thank you very much.
